# Lemneolemnanes A–D, Four Uncommon Sesquiterpenoids from the Soft Coral *Lemnalia* sp.

**DOI:** 10.3390/md22040145

**Published:** 2024-03-26

**Authors:** Yuan Zong, Tian-Yun Jin, Jun-Jie Yang, Kun-Ya Wang, Xing Shi, Yue Zhang, Ping-Lin Li

**Affiliations:** 1 Key Laboratory of Marine Drugs, Chinese Ministry of Education, School of Medicine and Pharmacy, Ocean University of China, Qingdao 266003, China; zongyuan@stu.ouc.edu.cn (Y.Z.); yangjunjie@stu.ouc.edu.cn (J.-J.Y.); sx79494@163.com (X.S.); zhangyue_00803@163.com (Y.Z.); 2 Laboratory of Marine Drugs and Biological Products, National Laboratory for Marine Science and Technology, Qingdao 266235, China; 3 Center for Marine Biotechnology and Biomedicine, Scripps Institution of Oceanography, University of California San Diego, La Jolla, CA 92093-0204, USA; t2jin@ucsd.edu; 4 State Key Laboratory of Bioactive Substance and Function of Natural Medicines, Institute of Materia Medica, Chinese Academy of Medical Sciences & Peking Union Medical College, Beijing 100050, China; phoebewky@163.com

**Keywords:** soft coral, *Lemnalia* sp., sesquiterpenoids, anti-Alzheimer’s disease activity

## Abstract

Four undescribed sesquiterpenoids, lemneolemnanes A–D (**1**–**4**), have been isolated from the marine soft coral *Lemnalia* sp. The absolute configurations of the stereogenic carbons of **1**–**4** were determined by single-crystal X-ray crystallographic analysis. Compounds **1** and **2** are epimers at C-3 and have an unusual skeleton with a formyl group on C-6. Compound **3** possesses an uncommonly rearranged carbon skeleton, while **4** has a 6/5/5 tricyclic system. Compound **1** showed significant anti-Alzheimer’s disease (AD) activity in a humanized *Caenorhabditis elegans* AD pathological model.

## 1. Introduction

Soft corals belonging to the genus *Lemnalia* have long been considered as a rich source of various terpenes [1], encompassing sesquiterpenes [2], diterpenes [3,4], and diterpenoid glycosides [5,6]. Sesquiterpenes, in particular, represent a prominent class of metabolites derived from *Lemnalia*, exhibiting diverse carbon skeletons, including ylangane-type [7], eremophilane-type [8], nardosinane-type [9,10,11], and neolemnane-type [12], among others. Typically, neolemnane sesquiterpenes have a 6/8 bicyclic skeleton, with the potential for producing rearranged skeletons [8,13]. In addition, these sesquiterpenoids with distinct skeletons exhibited a myriad of bioactivities, such as cytotoxic [7,14], anti-inflammatory [15,16], antimicrobial [17], antiviral [10], and neuroprotective activities [18]. In summary, the investigation of sesquiterpenes in soft corals of the genus *Lemnalia* is of significant interest due to their structural diversity and diverse bioactivities.

**Figure 1:**
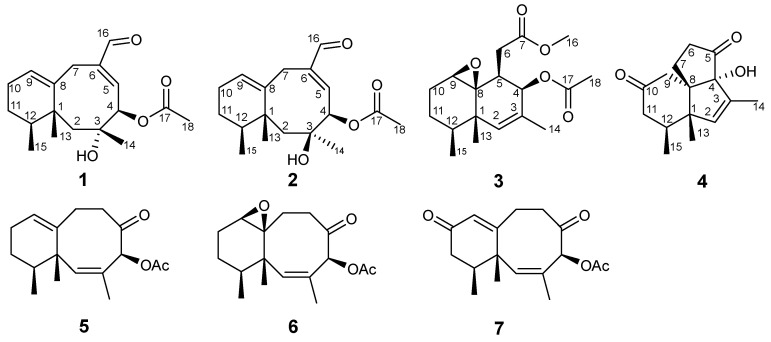
Structures of **1**–**7**.

In our ongoing quest for novel bioactive metabolites from soft corals, our investigation of the *Lemnalia* sp*.*, which was collected from the Xisha Islands, has led to the isolation of four unreported sesquiterpenoids, lemneolemnanes A–D (**1**–**4**), and three that have been previously reported: 4-acetoxy-2,8-neolemnadien-5-one (**5**) [19], paralemnolin G (**6**) [20], laevinone A (**7**) [14] (Figure 1). Compounds **1** and **2** are epimers at C-3, featuring an unusual carbon skeleton with a formyl group on C-6. Compound **3** possesses a rearranged 6/6 carbon skeleton, while **4** exhibits a rare skeleton with a 6/5/5 tricyclic system. Compound **4** is the second compound with the 6/5/5 tricyclic skeleton [13]. Herein, we report the isolation, structural elucidation, plausible biosynthetic pathways, and anti-AD activity of these compounds.

## 2. Results and Discussion

Compound **1** was isolated as colourless crystals. Its optical rotation value was [*α*]^25^_D_ + 57.8 (*c* 1.0, MeOH). The IR absorption peaks were found at 3415, 2808, 1728, and 1600 cm^−1^, indicating the presence of hydroxyl, aldehyde, ester carbonyl groups, and olefin, respectively. The molecular formula C_18_H_26_O_4_ was determined by HRESIMS *m/z* 307.1902 [M + H]^+^ (calcd for C_18_H_27_O_4_, 307.1904), implying six degrees of unsaturation. The ^13^C NMR spectrum (Table 1) displayed the presence of eighteen carbon signals which, in combination with the HSQC spectrum, can be classified as one aldehyde carbonyl (*δ*_C_ 194.0), one ester carbonyl (*δ*_C_ 170.8), two protonated sp^2^ (*δ*_C_ 149.7 and 129.0), two non-protonated sp^2^ (*δ*_C_ 142.8 and 139.4), one oxyquaternary sp^3^ (*δ*_C_ 76.8), one quaternary sp^3^ (*δ*_C_ 41.1), one oxymethine sp^3^ (*δ*_C_ 73.8), one methine sp^3^ (*δ*_C_ 34.4), four methylene sp^3^ (*δ*_C_ 43.6, 32.2, 27.3, and 26.3), and four methyl (*δ*_C_ 26.2, 22.8, 21.2, and 16.6) carbons. The ^1^H NMR spectrum (Table 1), in conjunction with the HSQC spectrum, displayed a singlet of the aldehydric proton at *δ*_H_ 9.35/*δ*_C_ 194.0, two olefinic protons at *δ*_H_ 6.21 (d, *J* = 5.7, 1.9 Hz)/*δ*_C_ 149.7 and *δ*_H_ 5.59 (dd, *J* = 5.7, 2.2 Hz)/*δ*_C_ 129.0, one oxymethine at *δ*_H_ 6.51 (d, *J* = 5.7 Hz)/*δ*_C_ 73.8, one methine at *δ*_H_ 2.47, m/*δ*_C_ 34.4, four pairs of methylene protons at *δ*_H_ 3.07 (d, *J* = 18.9 Hz) and 2.93 (d, *J* =18.9 Hz)/*δ*_C_ 32.2, *δ*_H_ 2.14, m and 2.04, m/*δ*_C_ 26.3, *δ*_H_ 1.87 (d, *J* = 15.8 Hz) and 1.42 (d, *J* = 15.8 Hz)/*δ*_C_ 43.6, and *δ*_H_ 1.49, m/*δ*_C_ 27.3, one methyl doublet at *δ*_H_ 0.98 (d, *J* = 6.7 Hz)/*δ*_C_ 16.6, and three methyl singlets at *δ*_H_ 2.15/*δ*_C_ 21.2, *δ*_H_ 1.14/*δ*_C_ 26.2, and *δ*_H_ 0.87/*δ*_C_ 22.8.

**Figure 2:**
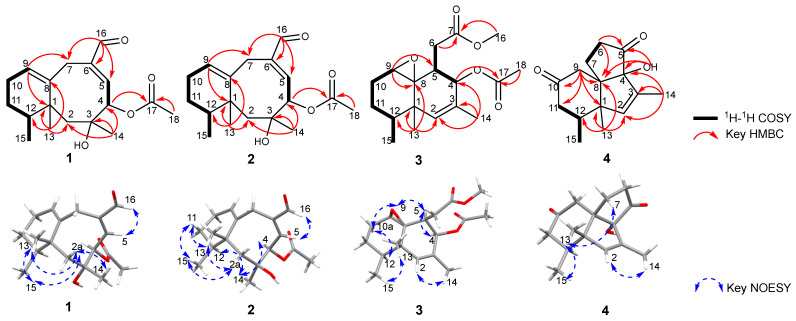
Key HMBC correlations (red arrows), ^1^H–^1^H COSY correlations (thick black line), and NOESY correlations (dotted arrow) of **1**–**4**.

The planar structure of **1** was elucidated by extensive analysis of ^1^H–^1^H COSY and HMBC spectra (Figure 2). The ^1^H–^1^H COSY correlations revealed the presence of two spin systems from H-4 to H-5 and H-9 to H_2_-10, H_2_-10 to H_2_-11, H_2_-11 to H-12, and H-12 to H_3_-15, while the HMBC spectrum showed correlations from H_3_-13 to C-1, C-2, C-8, and C-12; H_3_-14 to C-2, C-3, and C-4; H-16 to C-5, C-6, and C-7; and H-9 to C-1, C-7, C-10, and C-11, suggesting the presence of the 6/8 neolemnane sesquiterpene scaffold. The HMBC correlations from H-4 to C-17 and H_3_-18 to C-17 indicated the existence of an acetoxyl group on C-4, while the correlations from H-16 to C-5, C-6, and C-7 revealed the presence of a formyl substituent on C-6. The presence of a formyl group on a sesquiterpene skeleton is not common.

**Figure 3:**
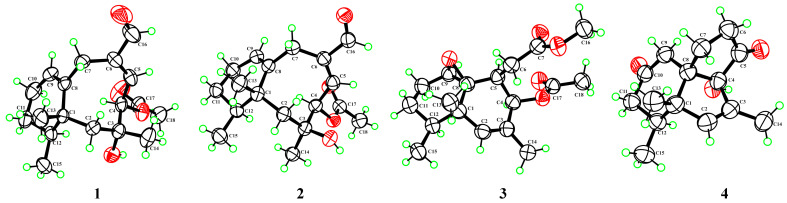
ORTEP diagrams of **1**–**4** (displacement ellipsoids are drawn at the 50% probability level).

The relative configuration of **1** was established by NOESY correlations (Figure 2). In the NOESY spectrum, H-5 displayed a correlation to H-16, confirming the *E*-configuration of the Δ^5,6^ double bond. NOESY cross-peaks from H_3_-13 to H_3_-15, H-2a, H_3_-15 to H-2a, and H_3_-14 to H-2a revealed that the three methyl groups were on the same face. The absence of a NOESY correlation from H-4 to H_3_-14 suggested that H-4 and H_3_-14 were on the opposite side. Thus, the relative configurations at C-1, C-3, C-4, and C-12 in **1** were established as 1R*, 3R*, 4R*, and 12S*. Finally, a suitable crystal of **1** was obtained for X-ray analysis using a diffractometer equipped with Cu Kα radiation. The Ortep diagram of **1** (Figure 3) shows the absolute configurations of C-1, C-3, C-4, and C-12 1*R*, 3*R*, 4*R*, and 12*S*, which were ascertained by a flack parameter of 0.2 (7) (Table S4).

Compound **2** was obtained as colourless crystals with an optical rotation, [*α*]^25^_D_ +1.6 (*c* 1.0, MeOH). The molecular formula of **2** was determined as C_18_H_26_O_4_ by HRESIMS *m/z* 307.1898 [M + H]^+^ (calcd for C_18_H_27_O_4_, 307.1904), which is the same as that of **1**. The ^1^H and ^13^C NMR spectra (Table 1) of **2** closely resembled those of **1**, with some chemical shift changes around the chiral C-3, suggesting that **2** could be a stereoisomer of **1**. Furthermore, the HMBC correlations and ^1^H–^1^H COSY cross-peaks (Figure 2) confirmed that the planar structure of **2** was the same as that of **1**.

In the NOESY spectrum (Figure 2), H-5 displayed a correlation to H-16, consistent with the *E*-configuration of the Δ^5,6^ double bond. The NOESY correlations from H_3_-13 to H-2a, H_2_-11, H_3_-15 to H-2a, H_2_-11, revealed that the two methyl groups were on the same face. In addition, the NOESY correlations from H-4 to H_3_-14 and H-12 to H_3_-14 indicated that H-4, H_3_-14, and H-12 were on the same side. Thus, the relative configuration at C-3 in **2** was different from that in **1**, and the relative configurations at C-1, C-3, C-4, and C-12 in **2** were established as 1R*, 3S*, 4R*, and 12S*. Ultimately, the absolute configurations at C-1, C-3, C-4 and C-12 in **2** were established as 1*R*, 3*S*, 4*R*, and 12*S* [Flack parameter of 0.02(6)] (Table S5) by the single-crystal X-ray diffraction experiment (Figure 3). Therefore, **1** and **2** are epimers at C-3.

Compound **3** was isolated as colourless crystals and its molecular formula C_18_H_26_O_5_ was determined by the HRESIMS *m/z* 345.1670 [M + Na]^+^ (calcd for C_18_H_26_O_5_Na, 345.1672), requiring six degrees of unsaturation. Compound **3** was levorotatory, with [*α*]^25^_D_ −10.8 (*c* 1.0, MeOH). The ^13^C NMR spectrum (Table 2) displayed the presence of eighteen carbon signals which, in combination with the HSQC spectrum, can be classified as two ester carbonyl (*δ*_C_ 173.5 and 170.3), one protonated sp^2^ (*δ*_C_ 132.5), one non-protonated sp^2^ (*δ*_C_ 128.7), one oxyquaternary sp^3^ (*δ*_C_ 63.1), one quaternary sp^3^ (*δ*_C_ 37.6), two oxymethine sp^3^ (*δ*_C_ 72.1 and 65.0), two methine sp^3^ (*δ*_C_ 43.7 and 39.5), three methylene sp^3^ (*δ*_C_ 32.6, 26.4, and 24.6), one methoxy (*δ*_C_ 51.7) and four methyl (*δ*_C_ 20.9, 18.8, 16.0, and 15.4) carbons. The ^1^H NMR spectrum (Table 2), in conjunction with the HSQC spectrum, displayed a singlet of the olefinic proton at *δ*_H_ 5.58/*δ*_C_ 149.7, two oxymethine at *δ*_H_ 5.68 (d, *J* = 5.3 Hz)/*δ*_C_ 72.1 and *δ*_H_ 3.15, m/*δ*_C_ 65.0, two methine at *δ*_H_ 2.24, m/*δ*_C_ 43.7 and *δ*_H_ 1.06, m/*δ*_C_ 39.5, three pairs of methylene protons at *δ*_H_ 2.60, m/*δ*_C_ 32.6, *δ*_H_ 2.03, m and 1.74, m/*δ*_C_ 26.4 and *δ*_H_ 1.30, m and 1.08, m/*δ*_C_ 24.6, one methyl doublet at *δ*_H_ 0.80 (d, *J* = 6.8 Hz)/*δ*_C_ 15.4, and four methyl singlets at *δ*_H_ 3.65/*δ*_C_ 51.7, *δ*_H_ 1.99/*δ*_C_ 20.9, *δ*_H_ 1.66/*δ*_C_ 18.8, and *δ*_H_ 0.92/*δ*_C_ 16.0.

The ^1^H–^1^H COSY correlations (Figure 2) from H-4 to H-5, H-5 to H-6, and H-9 to H_2_-10, H_2_-10 to H_2_-11, H_2_-11 to H-12, and H-12 to H_3_-15 revealed the presence of two spin systems, and together with the HMBC correlations (Figure 2) from H_3_-13 to C-1, C-2, C-8, and C-12; H_3_-14 to C-2, C-3, and C-4; and H-9 to C-5 and C-8, enabling the construction of an adecalin scaffold. In addition, the HMBC correlations from H-4 to C-17 and H_3_-18 to C-17 indicated the existence of an acetoxy group on C-4. The presence of a carbomethoxy on C-7 was supported by the HMBC correlations from H-6 to C-7 and H_3_-16 to C-7. The established planar structure of **3** has the same decalin scaffold as other nardosinane-type sesquiterpenoids, but differs from previously reported nardosinanes by the presence of the 2-ethoxy-2-methyl-2-oxoethyl group.

The relative configurations of **3** were partially established by the NOESY spectrum (Figure 2). The NOESY correlations from H-2 to H_3_-14 indicated the *Z*-configuration of the Δ^2,3^ double bond. The correlation from H_3_-13 to H_3_-15 in the NOESY spectrum indicated that Me-13 and Me-15 are cofacial. And the NOESY correlations from H-5 to H-4, H-9, and H-9 to H-10a, and H-12 to H-10a, showed that H-4, H-5, H-9, and H-12 are on the same side. Only the relative configuration of C-8 remained undetermined by the NOESY correlations. Since **3** could be obtained as a suitable crystal, the absolute configurations at C-1, C-4, C-5, C-8, C-9, and C-12 in **3** were determined as 1*S*, 4*R*, 5*S*, 8*S*, 9*R*, and 12*S* by a single-crystal X-ray diffraction experiment. Figure 3 shows the Ortep diagram of **3** with a Flack parameter of 0.04 (7) (Table S6).

Compound **4** was obtained as colourless crystals and is dextrorotatory, [*α*]^25^_D_ +181.9 (*c* 1.0, MeOH). The molecular formula of **3** was established as C_15_H_20_O_3_ by HRESIMS *m/z* 249.1485 [M + H]^+^ (calcd for C_15_H_21_O_3_, 249.1485), requiring six degrees of unsaturation. The ^13^C NMR spectrum (Table 3) displayed the presence of fifteen carbon signals which, in combination with the HSQC spectrum, can be classified as two ketone carbonyl (*δ*_C_ 217.8 and 211.0), one protonated sp^2^ (*δ*_C_ 141.0), one non-protonated sp^2^ (*δ*_C_ 137.6), one oxyquaternary sp^3^ (*δ*_C_ 93.6), two quaternary sp^3^ (*δ*_C_ 57.6 and 51.6), one methine sp^3^ (*δ*_C_ 39.4), four methylene sp^3^ (*δ*_C_ 43.9, 42.9, 33.7, and 30.3) and three methyl (*δ*_C_ 16.2, 12.1, and 11.0) carbons. The ^1^H NMR spectrum (Table 3), in conjunction with the HSQC spectrum, displayed one olefinic proton at *δ*_H_ 5.73 (d, *J* = 1.7 Hz)/*δ*_C_ 141.0, one methine at *δ*_H_ 2.28, m/*δ*_C_ 39.4, four pairs of methylene protons at *δ*_H_ 2.73 (d, *J* = 16.1 Hz) and 2.10, m/*δ*_C_ 42.9, *δ*_H_ 2.36, m and 2.22, m/*δ*_C_ 33.7, *δ*_H_ 2.22, m and 2.13, m/*δ*_C_ 43.9 and *δ*_H_ 2.00, m and 1.71, m/*δ*_C_ 30.3, two methyl doublets at *δ*_H_ 1.61 (d, *J* = 1.6 Hz)/*δ*_C_ 12.1 and *δ*_H_ 0.95 (d, *J* = 6.8 Hz)/*δ*_C_ 16.2, one methyl singlet at *δ*_H_ 1.04/*δ*_C_ 11.0, and a singlet of the hydroxyl proton *δ*_H_ 3.20. The ^1^H NMR signal at *δ*_H_ 5.73 (d, d, *J* = 1.7 Hz), along with the carbon signals at *δ*_C_ 141.0, 137.6, 217.8, and 211.0 indicated the presence of one trisubstituted double bond and two ketone groups, respectively. Thus, the remaining three degrees of unsaturation indicated the existence of a tricyclic system in **4**.

Firstly, the ^1^H–^1^H COSY correlations (Figure 2) from H_2_-11 to H-12 and from H-12 to H_3_-15 and the HMBC correlations (Figure 2) from H_3_-13 to C-1, C-2, C-8 and C-12; H_3_-14 to C-2, C-3, and C-4; and H-9 to C-1, C-8, C-10, and C-11 constructed a 6/5 ring system. Furthermore, the ^1^H−^1^H COSY correlations from H-6 to H-7, in combination with the HMBC correlations from H-6 to C-5 and C-8 and 4-OH to C-4, C-5, and C-8 indicated the presence of a cyclopentanone ring fused with the cyclohexene ring. Consequently, the planar structure of **4** was elucidated as a 6/5/5 ring system.

The NOESY spectrum (Figure 2) showed correlations from H_3_-13 to H_3_-15, suggesting that H_3_-13 and H_3_-15 are on the same face of the molecule. Moreover, the NOESY correlation between H_3_-13 and H_2_-7 revealed that H_3_-13 and H_2_-7 are on the same face. The absolute configurations of C-1, C-4, C-8, and C-12 in **4** were established as 1*S*, 4*R*, 8*S*, and 12*S* which was confirmed by a single-crystal X-ray diffraction experiment (Figure 3) with the Flack parameter of −0.1(2) (Table S7).

**Scheme 1:**
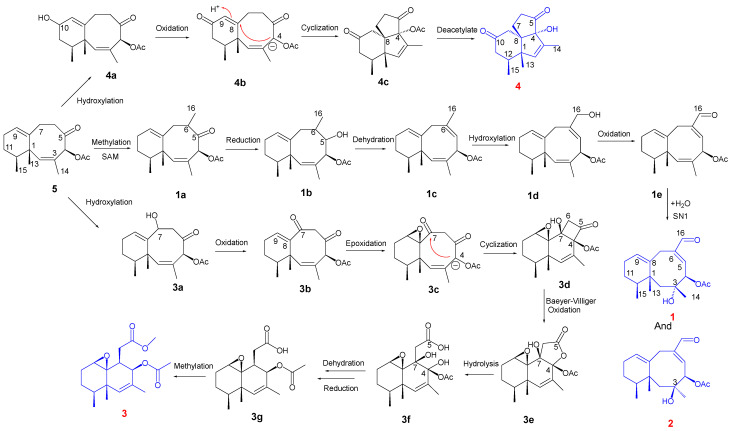
Proposed biosynthetic pathways of **1**–**4**.

Based on the structural features of **1**–**4**, we postulated that these compounds were originated from the same precursor **5** (Scheme 1). First, the methylation of **5** led to the formation of intermediate **1a**. Then, **1a** was converted to the intermediate **1c** by the reduction of the C-5 carbonyl of **1a** to the 5-hydroxyl group in **1b**, followed by dehydration of **1b** to give a double bond in **1c**. The hydroxylation of Me-16 of **1c** led to the formation of a primary alcohol in **1d** which, after oxidation, gave an aldehyde functionality at C-6 in **1e**. Finally, hydroxylation of the C2/C3 double bond in **1e** led to the formation of **1** and **2**. This was due to the fact that C-3 could form a more stable carbocation (tertiary) than C-2, and nucleophilic addition would occur via the SN1 mechanism, resulting in the formation of two isomers with opposite configurations. Compound **3** originated from the intermediate **3a**, a hydroxylation product of **5** at C-7. Compound **3a** was oxidized at C-7 to the form **3b**, which was converted to **3c** by the epoxidation of C-8/C-9. The dehydrogenation of **3c** at C-4 was cyclized at C-7/C-4 to form **3d**. Then, **3d** was converted to the lactone product **3e** via Baeyer–Villiger oxidation. Compound **3e** was converted to **3f** by hydrolysis, followed by the dehydration and reduction of **3f** to form the intermediate **3g**. Finally, the methylation of **3g** led to the formation of **3**, while in the proposed biosynthetic pathway of compound **4**, the precursor **4a** was the hydroxylation product of **5** at C-10. Then, **4a** was converted to the intermediate **4b** by the oxidation of C-10. The dehydrogenation of **4b** at C-4 induced subsequent cyclization of C-4/C-8 which led to the formation of the intermediate **4c**, which was converted to the form **4** by the deacetylation of C-4.

**Figure 4:**
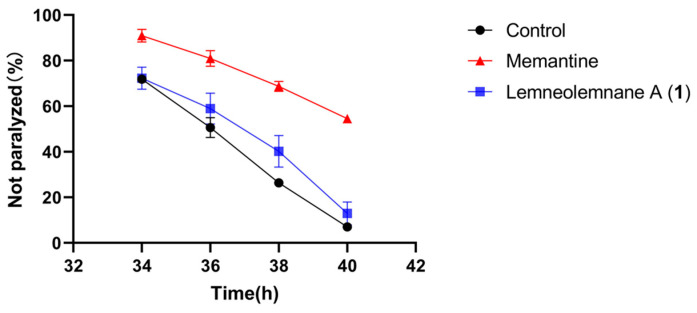
Anti-Alzheimer’s disease (AD) activity of **1** (worms were treated with 100 μM/L of **1**, 100 μM/L memantine was used as a positive control, and 0.1% dimethyl sulfoxide was used as a negative control).

Compounds **1**–**4** were first screened for cytotoxic and anti-inflammatory properties. However, none of the compounds showed significant anti-inflammatory activity in CuSO_4_-induced transgenic fluorescent zebrafish, or cytotoxic activity. Furthermore, **1**–**4** were evaluated for anti-AD activity using the transgenic *Caenorhabditis elegans* model. Memantine was used as a positive control due to its ability to reduce amyloid *β* (A*β*) peptide levels in human neuroblastoma cells, as well as to inhibit A*β* oligomer-induced synaptic loss [21], which effectively alleviates AD-like symptoms in worms [22]. Surprisingly, at a concentration of 100 μM/L, **1** significantly alleviated AD-like symptoms in the worm model (*p* < 0.05) (Figure 4). This result suggests that **1** has the potential to be a candidate for the development of an anti-AD agent based on its observed biological activity.

## 3. Materials and Methods

### 3.1. General Experimental Procedures

Melting points were measured on a microscopic melting point apparatus (Shanghai Zhuoguang Technology Company Limited, Shanghai, China). Optical rotations were measured on a Jasco P-1020 digital polarimeter (Jasco, Tokyo, Japan). The UV spectra were recorded on a Beckman DU640 spectrophotometer (Beckman Ltd., Shanghai, China). CD spectra were obtained on a Jasco J-810 spectropolarimeter (Jasco, Tokyo, Japan). IR (KBr) spectra were taken on a Nicolet NEXUS 470 spectrophotometer in KBr discs (Thermo Scientific, Beijing, China). NMR spectra were recorded using the Agilent 500 MHz (Agilent, Beijing, China), (^1^H, 500 MHz; ^13^C, 125 MHz) or the Bruker AVANCE NEO 400 (^1^H, 400 MHz; ^13^C, 100 MHz), (Bruker, Faellanden, Switzerland). The 7.26 ppm and 77.16 ppm resonances of CDCl_3_ were used as internal references for the ^1^H and ^13^C NMR spectra, respectively. HRESIMS spectra were measured on Micromass Q-Tof Ultima GLOBAL GAA076LC mass spectrometers (Autospec-Ultima-TOF, Waters, Shanghai, China). The crystallographic data were obtained on a Bruker APEX-II CCD diffractometer (Bruker, Beijing, China) equipped with graphite-monochromatized Cu K*α* radiation. Semi-preparative HPLC was performed using a Waters 1525 pump (Waters, Singapore) equipped with a 2998 photodiode array detector and a SilGreen C_18_ column (SilGreen, 10 × 250 mm, 5 μm). Silica gel (200–300 mesh and 300–400 mesh), (Qingdao Marine Chemical Factory, Qingdao, China) was used for column chromatography. Chiral HPLC analysis and resolution were conducted on a chiral analytical column (Daicel, Shanghai, China).

### 3.2. Animal Material

The soft coral *Lemnalia* sp. was collected from Xisha Island (Yagong Island) of the South China Sea in 2014, and was frozen immediately after collection. The specimen was identified by Prof Ping-Jyun Sung, Institute of Marine Biotechnology, National Museum of Marine Biology & Aquarium, Pingtung 944, Taiwan. The voucher specimen (No. xs-yg-12) was deposited at the State Key Laboratory of Marine Drugs, Ocean University of China, People’s Republic of China.

### 3.3. Extraction and Isolation

A frozen specimen of *Lemnalia* sp. (7.20 kg, wet weight) was thawed, homogenized, and then exhaustively extracted with CH_3_OH six times (3 days each time) at room temperature. The combined solutions were concentrated in vacuo and were subsequently desalted by redissolving in CH_3_OH to yield a residue (175.18 g). The crude extract was subjected to silica gel vacuum column chromatography and eluted with a gradient of petroleum ether/acetone (100:1 to 1:1, *v*/*v*) and subsequently eluted with a gradient of CH_2_Cl_2_/MeOH (10:1 to 1:1, *v*/*v*) to obtain sixteen fractions (Frs.1–16). Each fraction was detected by TLC. Fr.2 was subjected to silica gel vacuum column chromatography and eluted with petroleum ether/acetone, from 100:1 to 1:1, *v*/*v*, to give three subfractions Sfrs.2.1–2.3. Sfr.2.2 was purified by reversed-phase HPLC (OD-H, MeOH/H_2_O = 80/20, flow rate = 1.5 mL/min, I = 210 nm) to afford **5** (28.8 mg, t_R_ = 36 min). Fr.4 was subjected to silica gel vacuum column chromatography and eluted with petroleum ether/acetone, from 80:1 to 1:1, *v*/*v*, to give seven subfractions Sfrs.4.1–4.7. Sfr.4.5 was purified by reversed-phase HPLC (OD-H, MeOH/H_2_O = 70/30, flow rate = 1.5 mL/min, I = 210 nm) to afford mixtures of **3** and **6** (24.6 mg, t_R_ = 42 min). The mixture of **3** was purified with a chiral column (Daicel Chiral pack IC, n-hexane/isopropanol = 60:40, flow rate = 1.0 mL/min, I = 210 nm) to give **3** (11.9 mg, t_R_ = 36 min). Fr.7 was subjected to silica gel vacuum column chromatography and eluted with petroleum ether/acetone, from 50:1 to 1:1, *v*/*v*, to give five subfractions Sfrs.7.1–7.5. Sfr.7.2 was purified by reversed-phase HPLC (OD-H, MeCN/H_2_O = 50/50, flow rate = 1.5 mL/min, I = 320 nm) to afford **1** (7.1 mg, t_R_ = 72 min). Fr.9 was subjected to silica gel vacuum column chromatography and eluted with petroleum ether/acetone, from 30:1 to 1:1, *v*/*v*, to give four subfractions Sfrs.9.1–9.4. Sfr.9.4 was purified by reversed-phase HPLC (OD-H, MeOH/H_2_O = 60/40, flow rate = 1.5 mL/min, I = 210 nm) to afford **7** (19.7 mg, t_R_ = 45 min). Fr.11 was subjected to silica gel vacuum column chromatography and eluted with petroleum ether/acetone, from 20:1 to 1:1, *v*/*v*, to give six subfractions Sfrs.11.1–11.6. Sfr.11.1 was purified by reversed-phase HPLC (OD-H, MeCN/H_2_O = 30/70, flow rate = 1.5 mL/min, I = 210 nm) to afford **4** (20.7 mg, t_R_ = 57 min). Sfr.11.2 was purified by reversed-phase HPLC (OD-H, MeCN/H_2_O = 50/50, flow rate = 1.5 mL/min, I = 320 nm) to afford **2** (7.3 mg, t_R_ = 45 min).

Lemneolemnane A (**1**): colourless crystals, mp 157.9–159.5 °C; [*α*]^25^_D_ +57.8 (*c* 1.0, MeOH); CD (*c* 1.0, MeOH) = Δ*ε*197 + 51.50, Δ*ε*226–50.33, Δ*ε*260 + 17.29; UV (MeOH) *λ*_max_ (log *ε*) = 196 (1.45) nm, *λ*_max_ (log *ε*) = 213(0.91) nm, *λ*_max_ (log *ε*) = 230(1.42) nm; IR (KBr) *ν*_max_ = 3415, 2808, 1728, 1600 cm^−1^; HRESIMS *m/z* 307.1902 [M + H]^+^ (calcd for C_18_H_27_O_4_, 307.1904). For ^1^H NMR and ^13^C NMR data, see Table 1.

Lemneolemnane B (**2**): colourless crystals, mp 186.5–188.3 °C; [*α*]^25^_D_ +1.6 (*c* 1.0, MeOH); CD (*c* 0.5, MeOH) = Δ*ε*199 + 70.30, Δ*ε*225–83.68, Δ*ε*255 + 17.48; UV (MeOH) *λ*_max_ (log *ε*) = 196 (1.62) nm, *λ*_max_ (log *ε*) = 212(1.16) nm, *λ*_max_ (log *ε*) = 226(1.66) nm; IR (KBr) *ν*_max_ = 3431, 2831, 1726, 1599 cm^−1^; HRESIMS *m/z* 307.1898 [M + H]^+^ (calcd for C_18_H_27_O_4_, 307.1904). For ^1^H NMR and ^13^C NMR data, see Table 1.

Lemneolemnane C (**3**): colourless crystals, mp 135.6–137.9 °C; [*α*]^25^_D_ −10.8 (*c* 1.0, MeOH); CD (*c* 0.5, MeOH) = Δ*ε*201 + 18.60; UV (MeOH) λ_max_ (log *ε*) = 195 (1.99) nm, *λ*_max_ (log *ε*) = 193(0.96) nm; IR (KBr) *ν*_max_ = 1641 cm^−1^; HRESIMS *m/z* 323.1854 [M + H]^+^ (calcd for C_18_H_27_O_5_, 323.1853) and 345.1670 [M + Na]^+^ (calcd. for C_18_H_26_O_5_Na, 345.1672). For ^1^H NMR and ^13^C NMR data, see Table 2.

Lemneolemnane D (**4**): colourless crystals, mp 176.3–177.8 °C; [*α*]^25^_D_ +181.9 (*c* 1.0, MeOH); CD (*c* 0.5, MeOH) = Δ*ε*198 + 43.31, Δ*ε*231–35.02, Δ*ε*309 + 72.61; UV (MeOH) λ_max_ (log *ε*) = 193 (1.15) nm, λ_max_ (log *ε*) = 208 (0.78) nm, *λ*_max_ (log *ε*) = 218 (0.80) nm; IR (KBr) *ν*_max_ = 3415, 1716 cm^−1^; HRESIMS *m/z* 249.1485 [M + H]^+^ (calcd for C_15_H_21_O_3_, 249.1485) and 266.1751 [M + NH_4_]^+^ (calcd. for C_15_H_24_O_3_ N, 266.1751). For ^1^H NMR and ^13^C NMR data, see Table 3.

### 3.4. Anti-Alzheimer’s Disease Activity

The transgenic *Caenorhabditis elegans* strain CL4176 with genotype smg-1ts (myo-3/A*β*_1-42_ long 3′-untranslated region (UTR)) was obtained from the *Caenorhabditis* Genetics Center (CGC) (University of Minnesota, Minneapolis, MN, USA). Worms were routinely cultured on nematode growth medium (NGM) plates for 72 h to grow to the L3 stage at 16 °C, using *Escherichia coli* OP50 as the standard food resource. Throughout the bioactivity assays, 60~80 worm eggs were placed on NGM containing 100 μM/L of the test compounds, 100 μM/L memantine as a positive control, and 0.1% dimethyl sulfoxide solvent as a negative control. When the worms grew into L3 larvae, they were then transferred into another incubator which was set at 25 °C for another 32 h. Paralysed worms were observed and counted under a dissecting microscope every 2 h until all worms were paralysed. The anti-AD activity of the tested compound was indicated by the percentage of individual paralyzed worms in the tested worm population throughout the whole experiment in contrast with the negative control group. For the anti-AD activity assay, a log-rank survival test was used to compare the significance among treatments. *p* values at 0.05 or lower were considered statistically significant.

### 3.5. X-ray Crystallographic Analysis

X-ray crystallographic data were collected on a Bruker APEX-II CCD diffractometer. The crystal was kept at 150.0 K or 293.0 K during data collection. Using Olex2, the structures were solved with the SHELXT structure solution program using intrinsic phasing and refined with the SHELXL refinement package using least squares minimization.

## 4. Conclusions

Soft corals belonging to the genus *Lemnalia* have been recognized as a rich source of various bioactive secondary metabolites. Our continuing chemical investigation of the Xisha soft coral *Lemnalia* sp*.* resulted in the isolation of four unreported sesquiterpenoids, lemneolemnanes A–D (**1**–**4**), together with three previously described sesquiterpenoids **5**−**7**. Compounds **1** and **2** are epimers at C-3 that have an unusual skeleton with a formyl group on C-6. Based on the proposed biosynthetic pathways, **1** and **2** could be artifacts originating from a hydration of the C-2 and C-3 double bond of the octacyclic ring of the intermediate after formylation. Compound **3** features a decalin scaffold and a 2-ethoxy-2-methyl-2-oxoethyl group, while **4** possesses a 6/5/5 tricyclic system. Based on the structural characterization of lemneolemnanes A–D (**1**–**4**), we hypothesized their possible biosynthetic pathways, originating from **5**. Compound **1** showed significant anti-Alzheimer’s disease (AD) activity in a *Caenorhabditis elegans* model. The above research enriches chemical libraries of the soft corals *Lemnalia* sp*.* and provides some references in further exploration of potential biological activity. Meanwhile, the discovery of these rare molecular structures will provide new ideas.

## Figures and Tables

**Table 1:** ^1^H NMR (500 MHz) and ^13^C NMR (125 MHz) data for **1**–**2** in CDCl_3_.

NO.	1		2	
	*δ*_C_ Type	*δ*_H_ (*J* in Hz)	*δ*_C_ Type	*δ*_H_ (*J* in Hz)
1	41.1, C		40.7, C	
2a	43.6, CH_2_	1.87 d (15.8)	46.2, CH_2_	1.87 d (15.1)
2b		1.42 d (15.8)		1.61 d (15.1)
3	76.8, C		77.0, C	
4	73.8, CH	6.51 d (5.6)	73.7, CH	6.34 d (5.6)
5	149.7, CH	6.21 dt (5.6; 1.9)	150.2, CH	6.30 dt (5.6; 1.9)
6	142.8, C		142.6, C	
7a	32.2, CH_2_	3.07 d (18.9)	32.6, CH_2_	3.03 m
7b		2.93 d (18.9)		3.03 m
8	139.4, C		139.1, C	
9	129.0, CH	5.59 dd (5.7; 2.2)	129.4, CH	5.62 d (3.7)
10a	26.3, CH_2_	2.14 m	26.0, CH_2_	2.13 m
10b		2..04 m		2..03 m
11a	27.3, CH_2_	1.49 m	27.3, CH_2_	1.52 m
11b		1.49 m		1.52 m
12	34.4, CH	2.47 m	34.2, CH	2.00 m
13	22.8, CH_3_	0.87 s	22.9, CH_3_	0.92 s
14	26.2, CH_3_	1.14 s	27.2, CH_3_	1.44 s
15	16.6, CH_3_	0.98 d (6.7)	16.5, CH_3_	0.96 d (6.7)
16	194.0, CH	9.35 s	194.1, C	9.38 s
17	170.8, C		170.4, C	
18	21.2, CH_3_	2.15 s	21.1, CH_3_	2.16 s

**Table 2:** 1D and 2D NMR data for **3** in CDCl_3_.

NO.	3				
	*δ*_C_ ^a^ Type	*δ*_H_ ^b^ (*J* in Hz)	^1^H–^1^H COSY	HMBC	NOESY
1	37.6, C				
2	132.5, CH	5.58 s		C-1, C-4, C-8, C-12, C-13, C-14	H_3_-14
3	128.7, C				
4	72.1, CH	5.68 d (5.3)	H-5	C-2, C-3, C-5, C-6, C-17	H-5
5	43.7, CH	2.24 m	H-4, H-6	C-1, C-3, C-4, C-6, C-7, C-8, C-9	H-4, H-9
6a	32.6, CH_2_	2.60 m	H-5	C-4, C-5, C-7, C-8	
6b		2.60 m			
7	173.5, C				
8	63.1, C				
9	65.0, CH	3.15 m	H-10a	C-5, C-8, C-10, C-11	H-5, H-10a
10a	26.4, CH_2_	1.74 m	H-9, H_2_-11	C-8, C-9, C-11, C-12	H-9, H-12
10b		2.03 m			
11a	24.6, CH_2_	1.30 m	H_2_-10, H-12	C-1, C-9, C-10, C-12, C-15	
11b		1.08 m			
12	39.5, CH	1.06 m	H-11, H_3_-15	C-1, C-2, C-11, C-13, C-15	H-10a
13	16.0, CH_3_	0.92 s		C-1, C-2, C-8, C-12	H_3_-15
14	18.8, CH_3_	1.66 s		C-2, C-3, C-4	H-2
15	15.4, CH_3_	0.80 d (6.8)	H-12	C-1, C-11, C-12	H_3_-13
16	51.7, CH_3_	3.65 s		C-7	
17	170.3, C				
18	20.9, CH_3_	1.99 s		C-17	

^a^ Recorded at 125 MHz. ^b^ Recorded at 500 MHz.

**Table 3:** 1D and 2D NMR data for **4** in CDCl_3_.

NO.	4				
	*δ*_C_ ^a^ Type	*δ*_H_ ^b^ (*J* in Hz)	^1^H–^1^H COSY	HMBC	NOESY
1	51.6, C				
2	141.0, CH	5.73 d (1.7)		C-1, C-3, C-4, C-8, C-13, C-14	H_3_-13, H_3_-15
3	137.6, C				
4	93.6, C				
5	217.8, C				
6a	33.7, CH_2_	2.36 m	H-6	C-5, C-7, C-8	
6b		2.22 m			
7a	30.3, CH_2_	2.00 m	H-7	C-1, C-4, C-5, C-6, C-8, C-9	
7b		1.71 m			
8	57.6, C				
9a	42.9, CH_2_	2.73 d (16.1)		C-1, C-4, C-7, C-8, C-10, C-11	
9b		2.10 m			
10	211.0, C				
11a	43.9, CH_2_	2.22 m	H-12	C-1, C-10, C-12, C-15	
11b		2.13 m			
12	39.4, CH	2.28 m	H-11, H-15	C-1, C-2, C-10, C-11, C-13, C-15	
13	11.0, CH_3_	1.04 s		C-1, C-2, C-8, C-12	H-7, H_3_-15
14	12.1, CH_3_	1.61 d (1.6)		C-2, C-3, C-4	H_3_-14
15	16.2, CH_3_	0.95 d (6.8)	H-12	C-1, C-11, C-12	H_3_-13
OH		3.20 s		C-4, C-5, C-8	

^a^ Recorded at 125 MHz. ^b^ Recorded at 500 MHz.

## Data Availability

Data are contained within the article or Supplementary Materials.

## Supplementary Materials

The following supporting information can be downloaded at: https://www.mdpi.com/article/10.3390/md22040145/s1, Tables S1–S4: NMR data of **1**–**4**; Tables S5–S8 and Figures S1–S4: X-ray crystallographic analyses of **1**–**4**; Figures S5–S42: NMR spectra of **1**–**7**.
