# Supplementary material for: Lemneolemnanes A–D, Four Uncommon Sesquiterpenoids from the Soft Coral Lemnalia sp
Source: Mar Drugs. 2024 Mar 26;22(4):145. doi: 10.3390/md22040145 (PMC11051150; doi:10.3390/md22040145)
Supplement: Supplementary file 1 [file marinedrugs-22-00145-s001.zip › marinedrugs-2894645-supplementary.pdf]

Supporting Information

Lemneolemnanes A−D, Four Uncommon Sesquiterpenoids from the Soft Coral *Lemnalia* sp.

Yuan Zong 1,2, Tian-Yun Jin 3, Jun-Jie Yang 1,2, Kun-Ya Wang 4, Xing Shi 1,2, Yue Zhang 1,2 and Ping-Lin Li 1,2,*

1 Key Laboratory of Marine Drugs, Chinese Ministry of Education, School of Medicine and Pharmacy, Ocean University of China, Qingdao 266003, China; zongyuan@stu.ouc.edu.cn (Y.Z.); yangjunjie@stu.ouc.edu.cn (J.-J.Y.); sx79494@163.com (X.S.); zhangyue_00803@163.com (Y.Z.)

2 Laboratory of Marine Drugs and Biological Products, National Laboratory for Marine Science and Technology,
Qingdao 266235, China

3 Center for Marine Biotechnology and Biomedicine, Scripps Institution of Oceanography, University of California, San Diego La Jolla, CA 92093-0204, USA; t2jin@ucsd.edu

4 State Key Laboratory of Bioactive Substance and Function of Natural Medicines, Institute of Materia Medica, Chinese Academy of Medical Sciences & Peking Union Medical College, Beijing 100050, China; phoebewky@163.com

* Correspondence: lipinglin@ouc.edu.cn; Tel.: +86-532-8203-3054

Table

1. 1D and 2D NMR Data for **1–2**. 4

Table S1. 1D and 2D NMR Data for Lemneolemnane A (**1**) in $CDCl_3$. 4

Table S2. 1D and 2D NMR Data for Lemneolemnane B (**2**) in $CDCl_3$ 5

Table S3. ^{1}H NMR and ^{13}C NMR data for **5−7** in $CDCl_3$. 6

2. X-ray crystallographic analyses of **1–4**. 7

Table S4. X-ray diffraction analysis of Lemneolemnane A (**1**). 8

Table S5. X-ray diffraction analysis of Lemneolemnane B (**2**). 9

Table S6. X-ray diffraction analysis of Lemneolemnane C (**3**). 10

Table S7. X-ray diffraction analysis of Lemneolemnane D (**4**). 11

Figure S1. ORTEP diagrams of **1** (displacement ellipsoids are drawn at the 50% probability level). 12

Figure S2. ORTEP diagrams of **2** (displacement ellipsoids are drawn at the 50% probability level). 13

Figure S3. ORTEP diagrams of **3** (displacement ellipsoids are drawn at the 50% probability level). 14

Figure S4. ORTEP diagrams of **4** (displacement ellipsoids are drawn at the 50% probability level). 15

3. Anti-Alzheimer's disease activity. 16

Table S8. Data related to anti-Alzheimer's disease activity testing 16

4. The 1D and 2D NMR spectra of **1–4** 17

Figure S5. HRESIMS spectrum of compound **1**. 17

Figure S6. UV spectrum of compound **1**. 17

Figure S7. ^{1}H NMR spectrum of compound **1** in $CDCl_3$, 500MHz. 18

Figure S8. ^{13}C NMR spectrum of compound **1** in $CDCl_3$, 125MHz. 18

Figure S9. HSQC spectrum of compound **1** in $CDCl_3$, 500MHz. 19

Figure S10. HMBC NMR spectrum of compound **1** in $CDCl_3$, 500MHz. 19

Figure S11. ^{1}H-^{1}H COSY NMR spectrum of compound **1** in $CDCl_3$, 500MHz. 20

Figure S12. NOESY NMR spectrum of compound **1** in $CDCl_3$, 500MHz. 20

Figure S13. HRESIMS spectrum of compound **2**. 21

Figure S14. UV spectrum of compound **2**. 21

Figure S15. ^{1}H NMR spectrum of compound **2** in $CDCl_3$, 500MHz. 22

Figure S16. ^{13}C NMR spectrum of compound **2** in $CDCl_3$, 125MHz. 22

Figure S17. HSQC spectrum of compound **2** in $CDCl_3$, 500MHz. 23

Figure S18. HMBC NMR spectrum of compound **2** in $CDCl_3$, 500MHz. 23

Figure S19. ^{1}H-^{1}H COSY NMR spectrum of compound **2** in $CDCl_3$, 500MHz. 24

Figure S20. NOESY NMR spectrum of compound **2** in $CDCl_3$, 500MHz. 24

Figure S21. HRESIMS spectrum of compound **3**. 25
Figure S22. UV spectrum of compound **3**. 25
Figure S23. ^{1}H NMR spectrum of compound **3** in $CDCl_3$, 500MHz. 26
Figure S24. ^{13}C NMR spectrum of compound **3** in $CDCl_3$, 125MHz........ 26
Figure S25. HSQC spectrum of compound **3** in CDCl3, 500MHz. 27
Figure S26. HMBC NMR spectrum of compound **3** in $CDCl_3$, 500MHz. 27
Figure S27. ^{1}H-^{1}H COSY NMR spectrum of compound **3** in $CDCl_3$, 500MHz........ 28
Figure S28. NOESY NMR spectrum of compound **3** in $CDCl_3$, 500MHz. 28
Figure S29. HRESIMS spectrum of compound **4.** 29
Figure S30. UV spectrum of compound **4**. 29
Figure S31. ^{1}H NMR spectrum of compound **4** in $CDCl_3$, 500MHz. 30
Figure S32. ^{13}C NMR spectrum of compound **4** in $CDCl_3$, 125MHz........ 30
Figure S33. HSQC spectrum of compound **4** in $CDCl_3$, 500MHz. 31
Figure S34. HMBC NMR spectrum of compound **4** in $CDCl_3$, 500MHz. 31
Figure S35. ^{1}H-^{1}H COSY NMR spectrum of compound **4** in $CDCl_3$, 500MHz........ 32
Figure S36. NOESY NMR spectrum of compound **4** in $CDCl_3$, 500MHz. 32
Figure S37. ^{1}H NMR spectrum of compound **5** in $CDCl_3$, 500MHz. 33
Figure S38. ^{13}C NMR spectrum of compound **5** in $CDCl_3$, 125MHz........ 33
Figure S39. ^{1}H NMR spectrum of compound **6** in $CDCl_3$, 500MHz. 34
Figure S40. ^{13}C NMR spectrum of compound **6** in $CDCl_3$, 125MHz........ 34
Figure S41. ^{1}H NMR spectrum of compound **7** in $CDCl_3$, 400MHz. 35
Figure S42. ^{13}C NMR spectrum of compound **7** in $CDCl_3$, 100MHz........ 35

1. 1D and 2D NMR Data for 1–2.

Table S1. 1D and 2D NMR Data for Lemneolemnane A (**1**) in $CDCl_3$.

NO.	1				
	δ_C[a] type	δ_H[b] (J in Hz)	^1H–^1H COSY	HMBC	NOESY
1	41.1, C				
2a	43.6, CH_2	1.87 d (15.8)		C-1, C-3, C-4, C-8, C-12, C-13, C-14	H_3-13, H_3-14, H_3-15
2b		1.42 d (15.8)			
3	76.8, C				
4	73.8, CH	6.51 d (5.7)	H-5	C-3, C-5, C-6, C-14, C-17	
5	149.7, CH	6.21 dt (5.6; 1.9)	H-4	C-4, C-6, C-7, C-16	H-16
6	142.8, C				
7a	32.2, CH_2	3.07 d (18.9)		C-1, C-5, C-6, C-8, C-9, C-16	
7b		2.93 d (18.9)			
8	139.4, C				
9	129.0, CH	5.59 dd (5.7; 2.2)	H_2-10	C-1, C-7, C-10, C-11	
10a	26.3, CH_2	2.14 m	H-9, H_2-11	C-8, C-9, C-11	
10b		2..04 m			
11a	27.3, CH_2	1.49 m	H_2-10, H-12	C-1, C-9, C-10, C-12, C-15	
11b		1.49 m			
12	34.4, CH	2.47 m	H_2-11, H_3-15	C-1, C-10, C-11, C-13, C-15	
13	22.8, CH_3	0.87 s		C-1, C-2, C-8, C-12	H-2a, H_3-15
14	26.2, CH_3	1.14 s		C-2, C-3, C-4	H-2a
15	16.6, CH_3	0.98 d (6.7)	H-12	C-1, C-11, C-12	H-2a, H_3-13
16	194.0, CH	9.35 s		C-5, C-6, C-7	H-5
17	170.8, C				
18	21.2, CH_3	2.15 s		C-17	

[a] Recorded at 125 MHz. [b] Recorded at 500 MHz.

Table S2. 1D and 2D NMR Data for Lemneolemnane B (**2**) in $CDCl_3$.

NO.	2				
	δ_C[a] type	δ_H[b] (*J* in Hz)	^1H–^1H COSY	HMBC	NOESY
1	40.7, C				
2a	46.2, CH_2	1.87 d (15.1)		C-1, C-3, C-4, C-8, C-12, C-13, C-14	H_3-13, H_3-15
2b		1.61 d (15.1)			
3	77.0, C				
4	73.7, CH	6.34 d (5.6)	H-5	C-5, C-6, C-17	H_3-14
5	150.2, CH	6.30 dt (5.6; 1.9)	H-4	C-4, C-6, C-7, C-16	H-16
6	142.6, C				
7a	32.6, CH_2	3.03 m		C-1, C-5, C-6, C-8, C-9, C-16	
7b		3.03 m			
8	139.1, C				
9	129.4, CH	5.62 d (3.7)	H_2-10	C-1, C-7, C-10, C-11	
10a	26.0, CH_2	2.13 m	H-9, H_2-11	C-8, C-9, C-11	
10b		2..03 m			
11a	27.3, CH_2	1.52 m	H_2-10, H-12	C-1, C-9, C-10, C-12, C-15	H_3-13, H_3-15
11b		1.52 m			
12	34.2, CH	2.00 m	H_2-11, H_3-15	C-1, C-10, C-11, C-13, C-15	H_3-14
13	22.9, CH_3	0.92 s		C-1, C-2, C-8, C-12	H-2a, H-11
14	27.2, CH_3	1.44 s		C-2, C-3, C-4	H-12, H-4
15	16.5, CH_3	0.96 d (6.7)	H-12	C-1, C-11, C-12	H-2a, H-11
16	194.1, C	9.38 s		C-5, C-6, C-7	H-5
17	170.4, C				
18	21.1, CH_3	2.16 s		C-17	

[a] Recorded at 125 MHz. [b] Recorded at 500 MHz.

Table S3. ^{1}H NMR and ^{13}C NMR data for **5–7** in $CDCl_3$.

NO.	5		6		7	
	δ_C[a] type	δ_H[b] (*J* in Hz)	δ_C[a] type	δ_H[b] (*J* in Hz)	δ_C[c] type	δ_H[d] (*J* in Hz)
**1**	43.8, C		42.3, C		45.1, C	
**2**	139.6, CH	5.47 s	142.1, CH	5.71 m	136.9, CH	5.55 s
**3**	126.4, C		126.7, C		129.0, C	
**4**	76.4, CH	6.41 s	77.1, CH	6.16 s	76.1, CH	5.98 s
**5**	203.2, C		201.5, C		200.6, C	
**6**	44.5, CH_2	2.61 m	40.7, CH_2	2.53 m	43.4, CH_2	2.74 m
**7**	28.5, CH_2	2.08 m	32.8, CH_2	2.34 m	29.5, CH_2	2.63 m
**8**	144.8, C		66.2, C		173.0, C	
**9**	124.6 CH	5.68 m	59.7, CH	3.47 d (2.7)	127.6, CH	6.13 s
**10**	25.4, CH_2	2.16 m	25.7, CH_2	2.12 m; 1.92 m	197.7, C	
**11**	27.2, CH_2	1.53 m	24.3, CH_2	1.38 m; 1.14 m	42.1, CH_2	2.38 m
**12**	39.2, CH	1.90 m	40.1, CH	1.52 m	39.1, CH	2.54 m
**13**	22.7, CH_3	1.00 s	18.8, CH_3	1.10 s	20.6, CH_3	1.21 s
**14**	18.4, CH_3	1.67 d (1.6)	17.7, CH_3	1.69 d (1.5)	18.1, CH_3	1.75 s
**15**	17.1, CH_3	0.93 d (7.0)	17.2, CH_3	0.84 d (6.8)	16.9, CH_3	1.07 d (8.4)
**OAc**	170.4, C		170.4, C		170.0, C	
**OAc**	20.7, CH_3	2.09 s	20.7, CH_3	2.15 s	20.5, CH_3	2.11 s

[a] Recorded at 125 MHz. [b] Recorded at 500 MHz. [c] Recorded at 100 MHz. [d] Recorded at 400 MHz.

2. X-ray crystallographic analyses of 1–4.

Lemneolemnane A (**1**) was obtained as colorless crystal from a methanol solvent system using the vapor diffusion method. Crystallographic data for **1** in this article have been deposited at the Cambridge Crystallographic Data Centre as supplementary publication number 2311688. The data can be obtained via www.ccdc.cam.ac.uk/products/csd/request.

Lemneolemnane B (**2**) was obtained as colorless crystal from a methanol solvent system using the vapor diffusion method. Crystallographic data for **2** in this article have been deposited at the Cambridge Crystallographic Data Centre as supplementary publication number 2311689. The data can be obtained via www.ccdc.cam.ac.uk/products/csd/request.

Lemneolemnane C (**3**) was obtained as colorless crystal from a methanol solvent system using the vapor diffusion method. Crystallographic data for **3** in this article have been deposited at the Cambridge Crystallographic Data Centre as supplementary publication number 2311686. The data can be obtained via www.ccdc.cam.ac.uk/products/csd/request.

Lemneolemnane D (**4**) was obtained as colorless crystal from a methanol solvent system using the vapor diffusion method. Crystallographic data for **4** in this article have been deposited at the Cambridge Crystallographic Data Centre as supplementary publication number 2311690. The data can be obtained via www.ccdc.cam.ac.uk/products/csd/request.

Table S4. X-ray diffraction analysis of Lemneolemnane A (**1**).

Empirical formula	$C_{18}H_{26}O_4$
Formula weight	306.39
Temperature/K	293(2)
Crystal system	orthorhombic
Space group	$P2_12_12_1$
a/Å	7.5112(3)
b/Å	8.4632(3)
c/Å	26.8781(9)
α/°	90
β/°	90
γ/°	90
Volume/Å3	1708.61(11)
Z	4
ϱcalcg/cm3	1.191
μ/mm-1	0.667
F (000)	664.0
Crystal size/mm3	0.2 × 0.15 × 0.1
Radiation	CuKα (λ = 1.54184)
2Θ range for data collection/°	6.578 to 142.1
Index ranges	-4 ⩽ h ⩽ 9, -9 ⩽ k ⩽ 10, -32 ⩽ l ⩽ 14
Reflections collected	4136
Independent reflections	2700 [R_{int} = 0.0512, R_{sigma} = 0.0656]
Data/restraints/parameters	2700/0/204
Goodness-of-fit on F2	1.039
Final R indexes [I>=2σ (I)]	R_1 = 0.0615, wR_2 = 0.1931
Final R indexes [all data]	R_1 = 0.0831, wR_2 = 0.2352
Largest diff. peak/hole / e Å-3	0.27/-0.23
Flack parameter	0.2(7)

Table S5. X-ray diffraction analysis of Lemneolemnane B (**2**).

Empirical formula	$C_{18}H_{26}O_4$
Formula weight	306.39
Temperature/K	150.00
Crystal system	orthorhombic
Space group	$P2_12_12_1$
a/Å	9.1612(4)
b/Å	12.9171(6)
c/Å	13.6650(6)
α/°	90
β/°	90
γ/°	90
Volume/Å3	1617.06(13)
Z	4
ϱcalcg/cm3	1.258
μ/mm-1	0.705
F (000)	664.0
Crystal size/mm3	0.2 × 0.15 × 0.1
Radiation	CuKα (λ = 1.54178)
2Θ range for data collection/°	9.422 to 149.678
Index ranges	-11 ≤ h ≤ 11, -14 ≤ k ≤ 16, -16 ≤ l ≤ 14
Reflections collected	8486
Independent reflections	3192 [R_{int} = 0.0250, R_{sigma} = 0.0269]
Data/restraints/parameters	3192/0/204
Goodness-of-fit on F2	1.132
Final R indexes [I>=2σ (I)]	R_1 = 0.0606, wR_2 = 0.1444
Final R indexes [all data]	R_1 = 0.0607, wR_2 = 0.1446
Largest diff. peak/hole / e Å-3	0.32/-0.20
Flack parameter	0.02(6)

Table S6. X-ray diffraction analysis of Lemneolemnane C (**3**).

Empirical formula	$C_{18}H_{26}O_5$
Formula weight	322.39
Temperature/K	150.00
Crystal system	orthorhombic
Space group	$P2_12_12_1$
a/Å	8.3795(2)
b/Å	8.5769(3)
c/Å	47.3938(14)
α/°	90
β/°	90
γ/°	90
Volume/Å3	3406.20(18)
Z	8
ϱcalcg/cm3	1.257
μ/mm-1	0.741
F (000)	1392.0
Crystal size/mm3	0.2 × 0.15 × 0.1
Radiation	CuKα (λ = 1.54178)
2Θ range for data collection/°	7.46 to 149.254
Index ranges	-9 ≤ h ≤ 10, -10 ≤ k ≤ 10, -58 ≤ l ≤ 59
Reflections collected	23967
Independent reflections	6894 [R_{int} = 0.0406, R_{sigma} = 0.0425]
Data/restraints/parameters	6894/0/425
Goodness-of-fit on F2	1.095
Final R indexes [I>=2σ (I)]	R_1 = 0.0945, wR_2 = 0.2733
Final R indexes [all data]	R_1 = 0.0963, wR_2 = 0.2765
Largest diff. peak/hole / e Å-3	0.39/-0.35
Flack parameter	0.21(6)

Table S7. X-ray diffraction analysis of Lemneolemnane D (**4**).

Empirical formula	$C_{15}H_{20}O_3$
Formula weight	248.31
Temperature/K	150.00
Crystal system	monoclinic
Space group	$P2_1$
a/Å	7.4946(3)
b/Å	6.8964(2)
c/Å	12.8420(4)
α/°	90
β/°	102.246(2)
γ/°	90
Volume/Å3	648.65(4)
Z	2
ϱcalcg/cm3	1.271
μ/mm-1	0.701
F (000)	268.0
Crystal size/mm3	0.2 × 0.15 × 0.1
Radiation	CuKα (λ = 1.54178)
2Θ range for data collection/°	7.044 to 149.35
Index ranges	-9 ⩽ h ⩽ 9, -6 ⩽ k ⩽ 8, -16 ⩽ l ⩽ 16
Reflections collected	5883
Independent reflections	2326 [R_{int} = 0.0358, R_{sigma} = 0.0363]
Data/restraints/parameters	2326/1/167
Goodness-of-fit on F2	1.059
Final R indexes [I>=2σ (I)]	R_1 = 0.0576, wR_2 = 0.1455
Final R indexes [all data]	R_1 = 0.0613, wR_2 = 0.1502
Largest diff. peak/hole / e Å-3	0.29/-0.17
Flack parameter	-0.1(2)

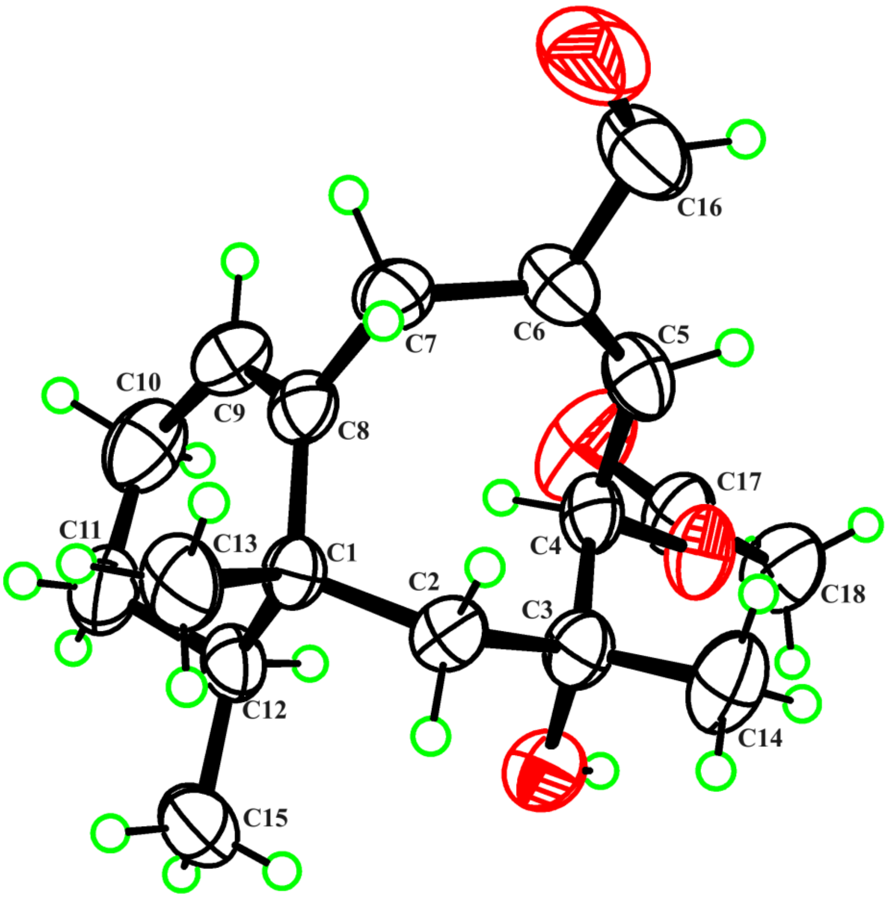

Figure S1. ORTEP diagrams of **1** (displacement ellipsoids are drawn at the 50% probability level).

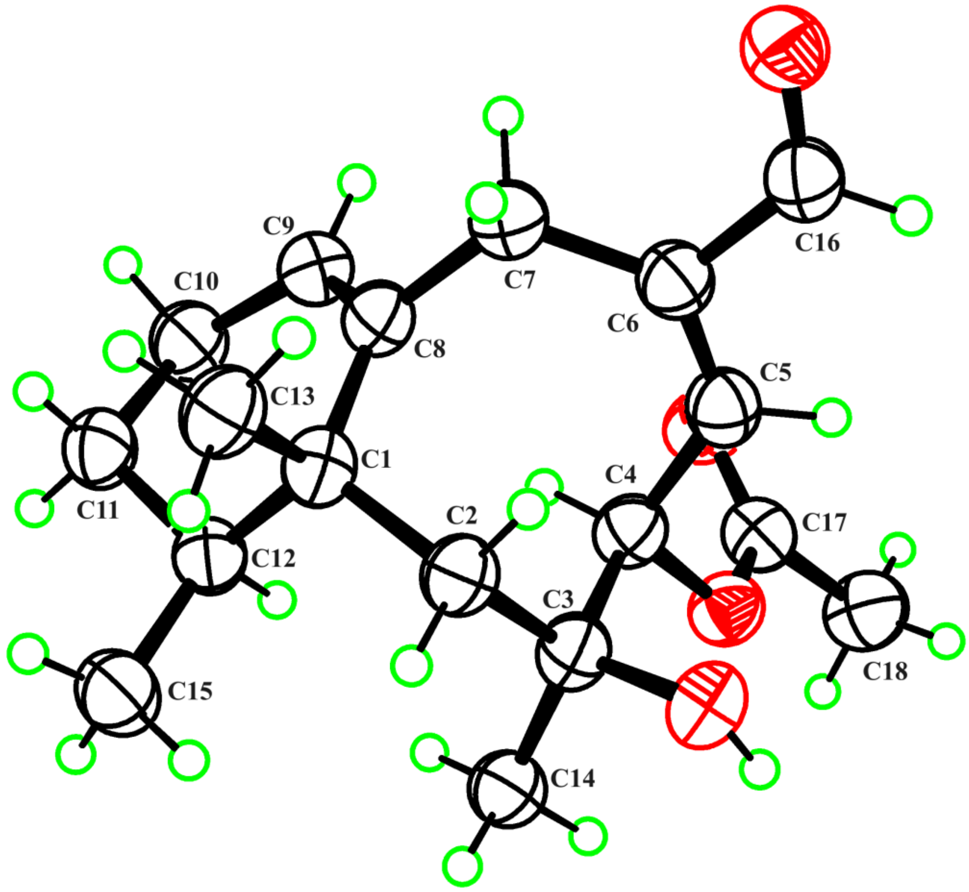

Figure S2. ORTEP diagrams of **2** (displacement ellipsoids are drawn at the 50% probability level).

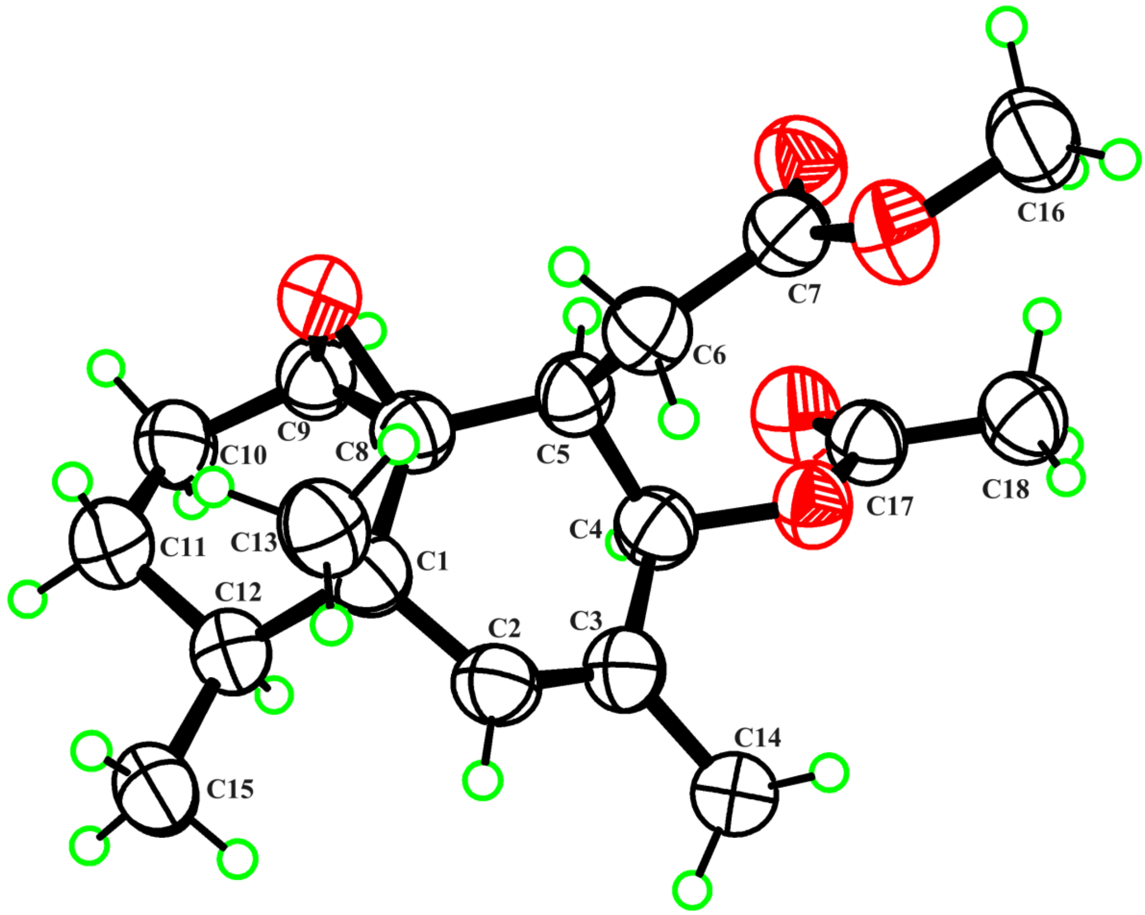

Figure S3. ORTEP diagrams of **3** (displacement ellipsoids are drawn at the 50% probability level).

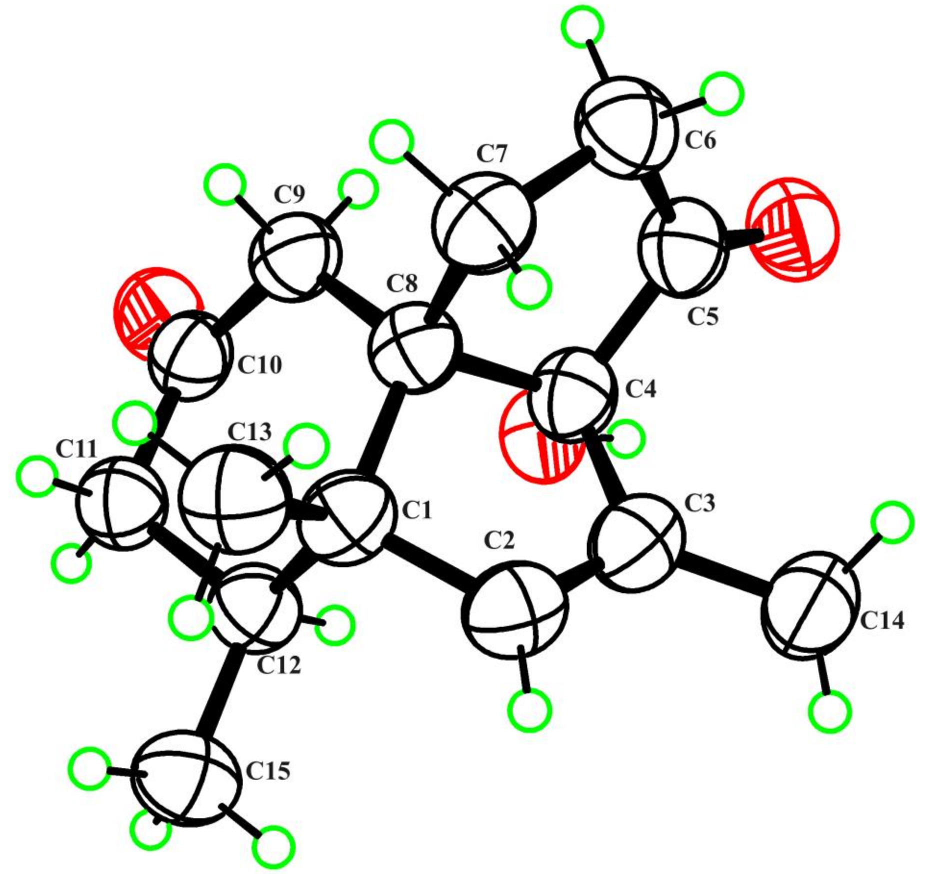

Figure S4. ORTEP diagrams of **4** (displacement ellipsoids are drawn at the 50% probability level).

3. Anti-Alzheimer's disease activity.

Table S8. Data related to anti-Alzheimer's disease activity testing

Time (h)	Control	Control	Control	Memantine	Memantine	Memantine	Compound **1**	Compound **1**	Compound **1**
34	12	10	13	2	4	3	9	7	8
36	9	6	12	4	4	2	4	3	6
38	13	9	8	5	3	4	2	3	5
40	8	7	9	4	5	5	3	4	5
Number of remaining unparalyzed	3	2	4	18	19	17	12	15	14
Total number	45	34	46	33	35	31	30	32	38
Time (h)	Percentage of survivors								
34	73.33	70.59	71.74	93.94	88.57	90.32	70.00	78.13	78.95
36	53.33	52.94	45.65	81.82	77.14	83.87	56.67	68.75	63.16
38	24.44	26.47	28.26	66.67	68.57	70.97	50.00	59.38	50.00
40	6.67	5.88	8.70	54.55	54.29	54.84	40.00	46.88	36.84
Average survival rate (MEAN) Standard deviation(SD)	Control	MEAN	SD	Memantine	MEAN	SD	Compound **1**	MEAN	SD
		71.89	1.38		90.94	2.74		75.69	4.95
		50.64	4.33		80.94	3.45		62.86	6.05
		26.39	1.91		68.74	2.16		53.13	5.41
		7.08	1.45		54.56	0.28		41.24	5.13

4. The 1D and 2D NMR spectra of 1–4

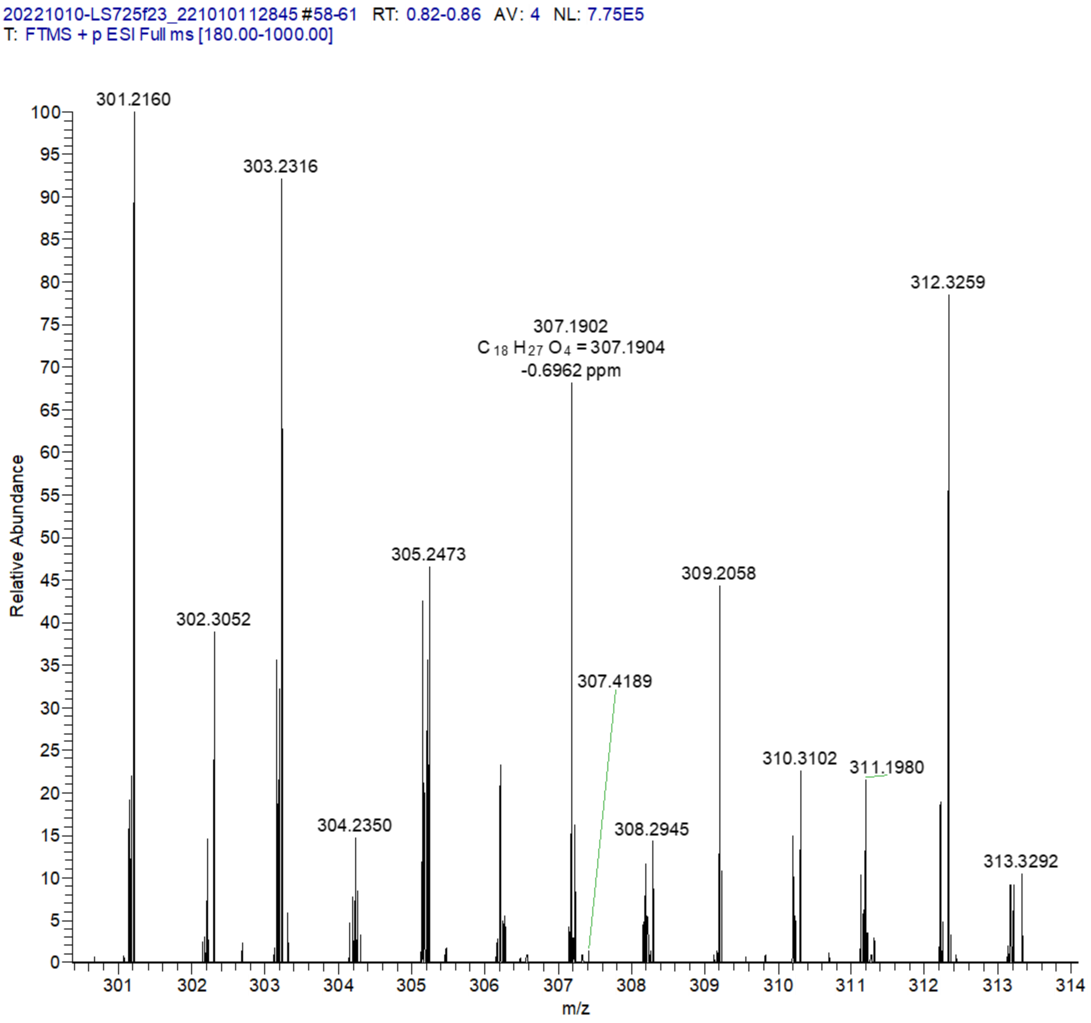

Figure S5. HRESIMS spectrum of compound **1**.

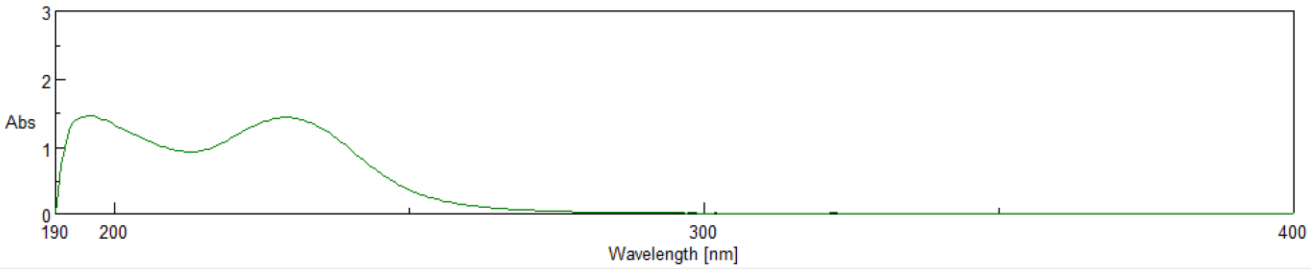

Figure S6. UV spectrum of compound **1**.

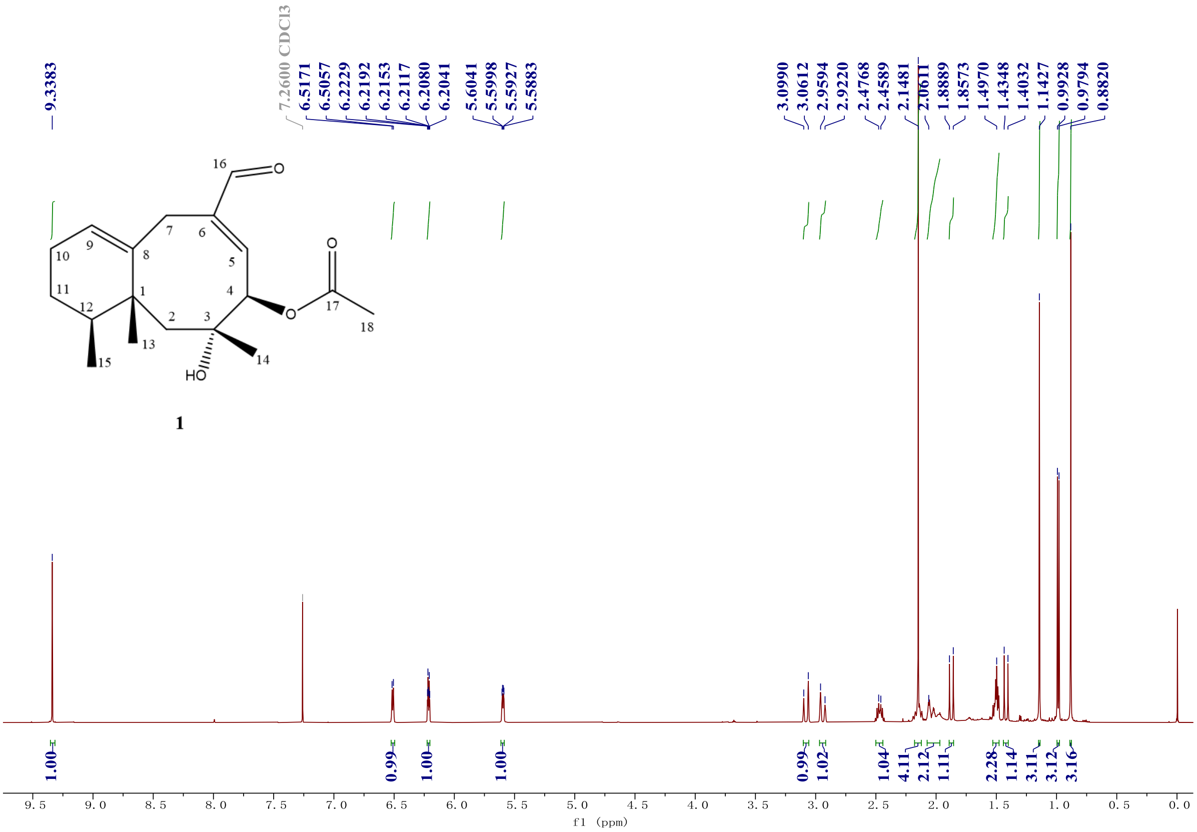

Figure S7. ¹H NMR spectrum of compound **1** in $CDCl_3$, 500MHz.

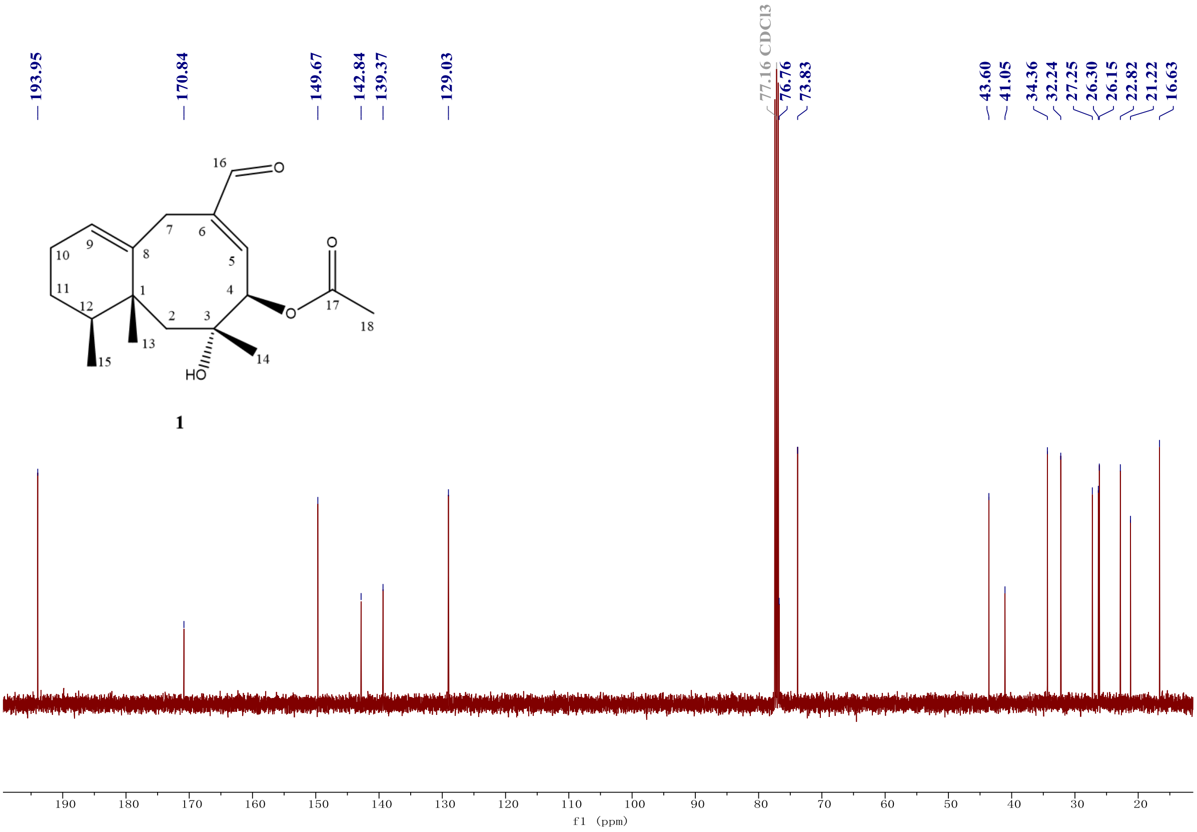

Figure S8. ¹³C NMR spectrum of compound **1** in $CDCl_3$, 125MHz.

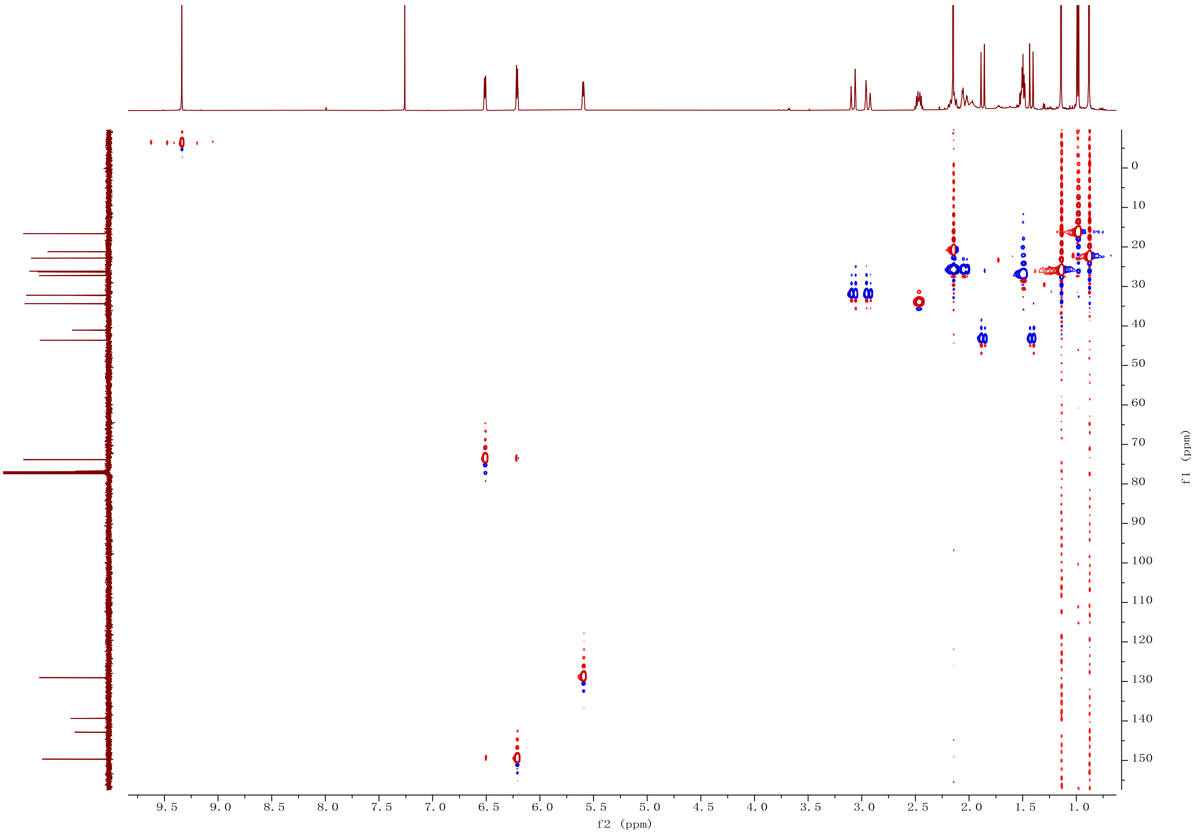

Figure S9. HSQC spectrum of compound **1** in $CDCl_3$, 500MHz.

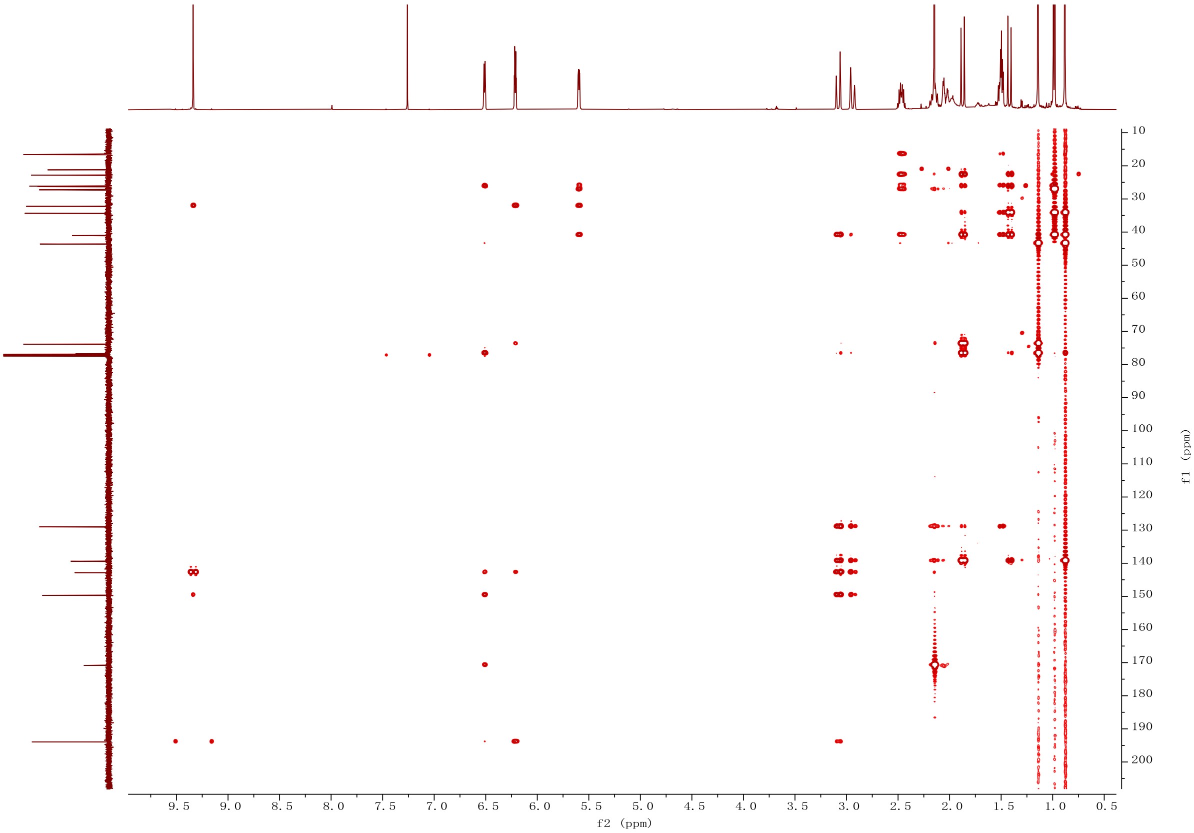

Figure S10. HMBC NMR spectrum of compound **1** in $CDCl_3$, 500MHz.

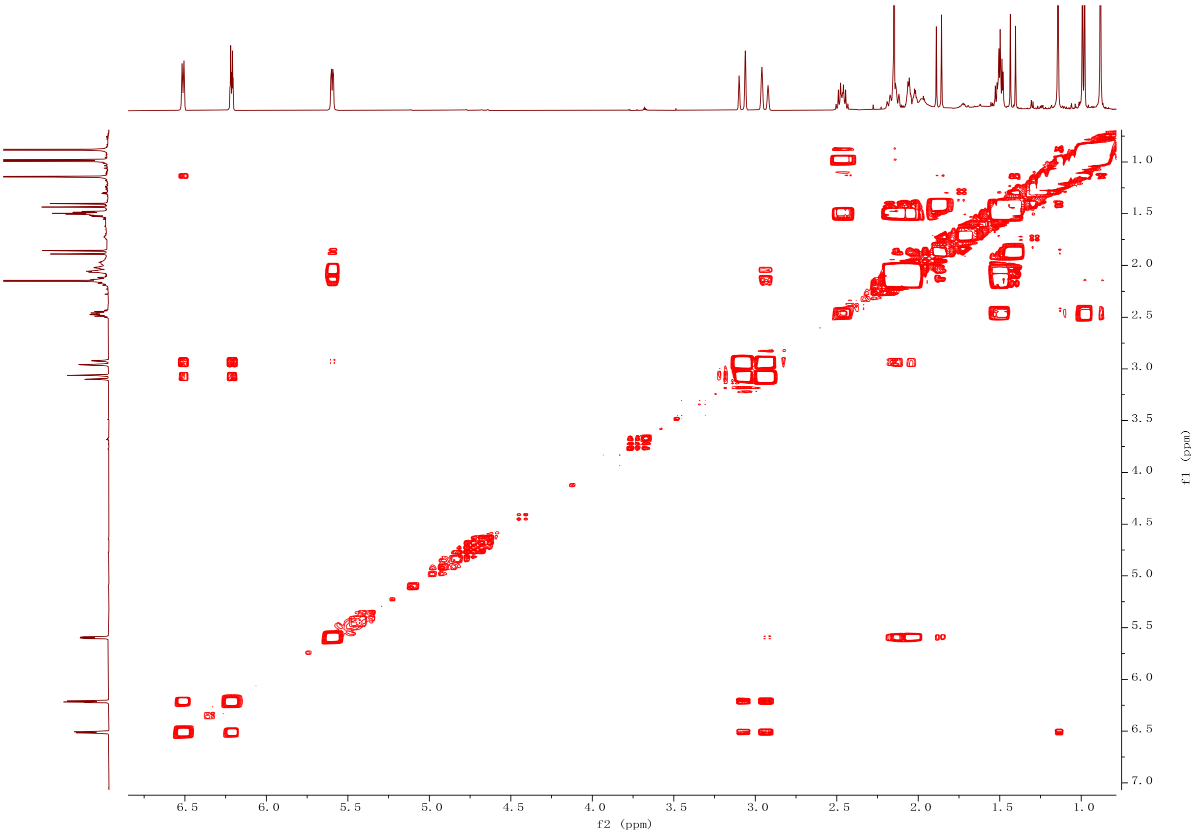

Figure S11. ^{1}H-^{1}H COSY NMR spectrum of compound **1** in $CDCl_3$, 500MHz.

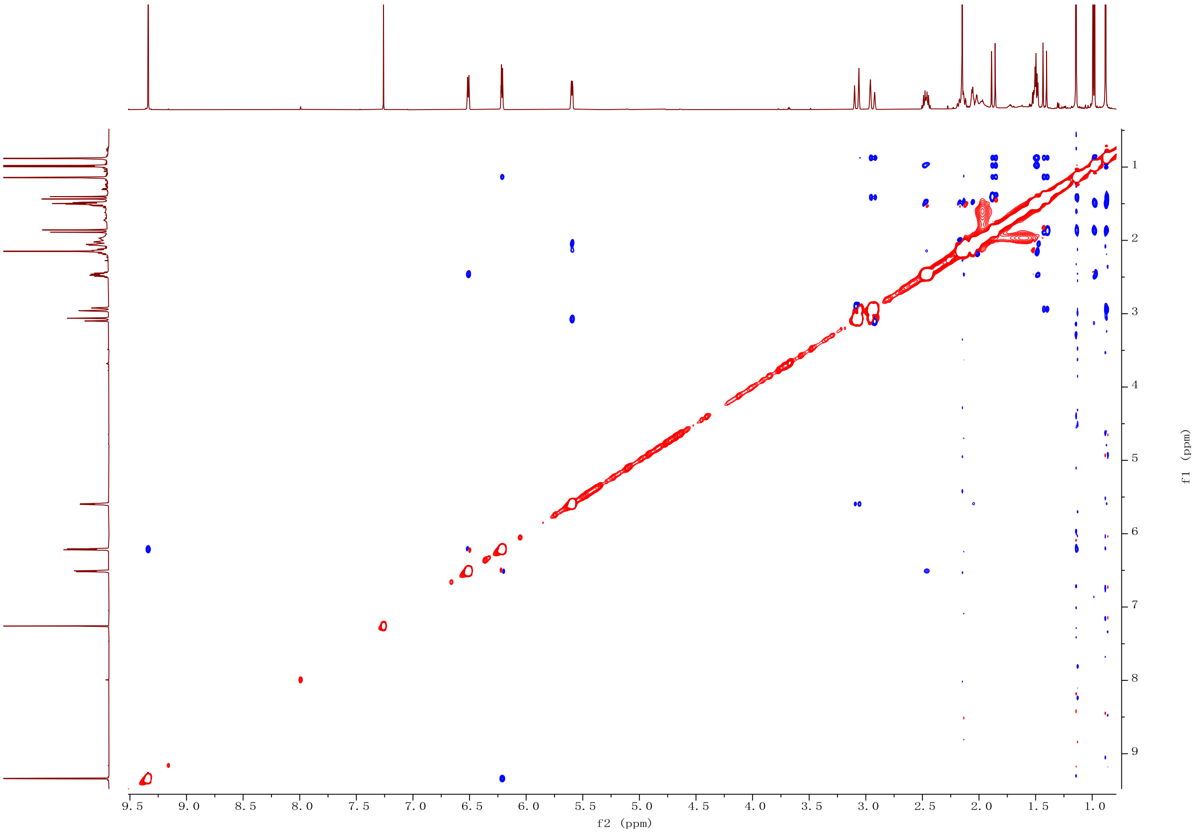

Figure S12. NOESY NMR spectrum of compound **1** in $CDCl_3$, 500MHz.

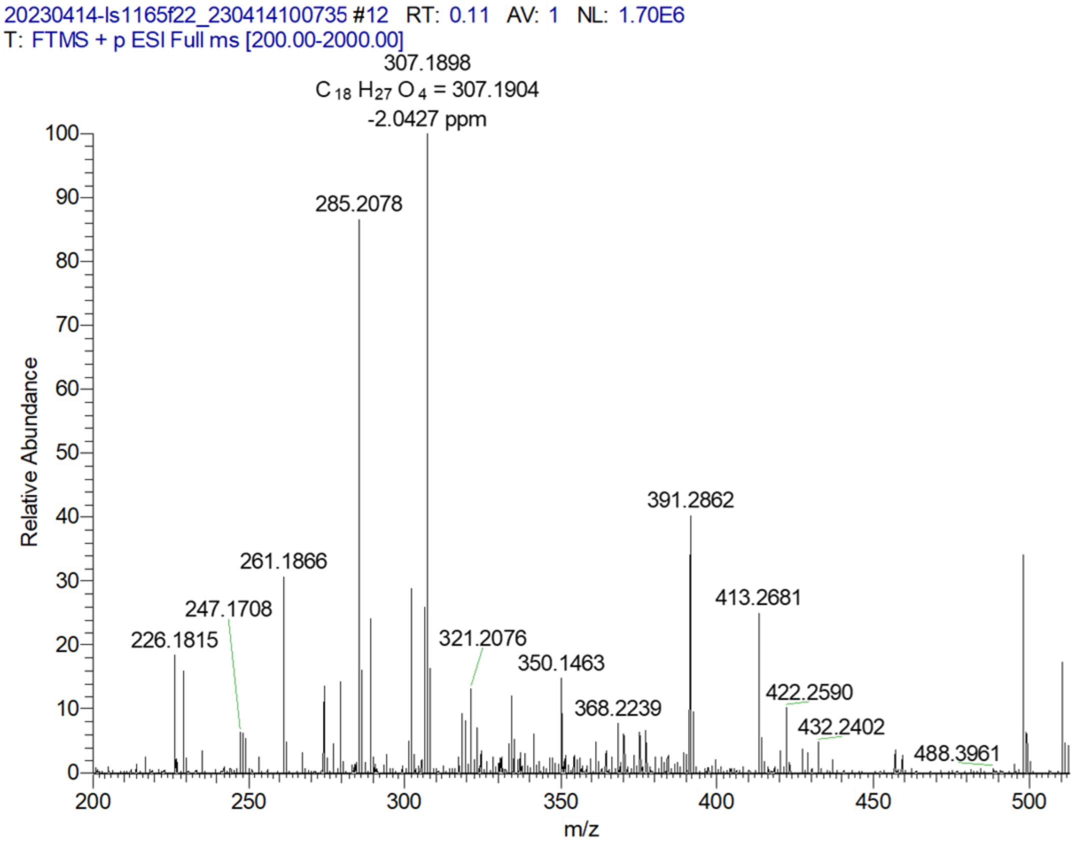

Figure S13. HRESIMS spectrum of compound **2**.

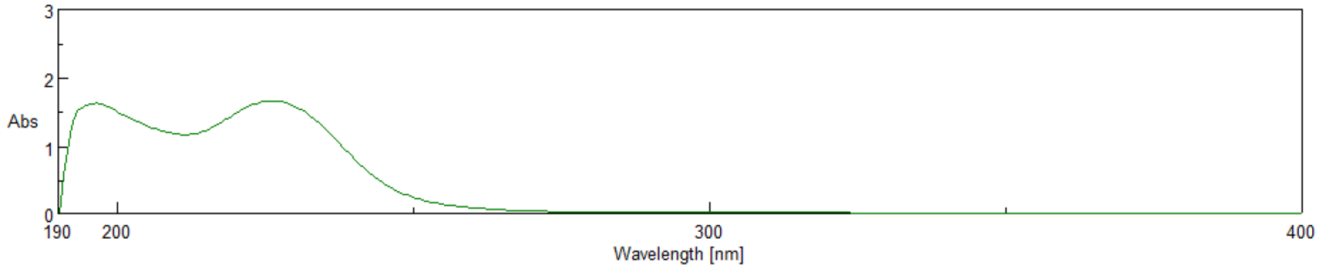

Figure S14. UV spectrum of compound **2**.

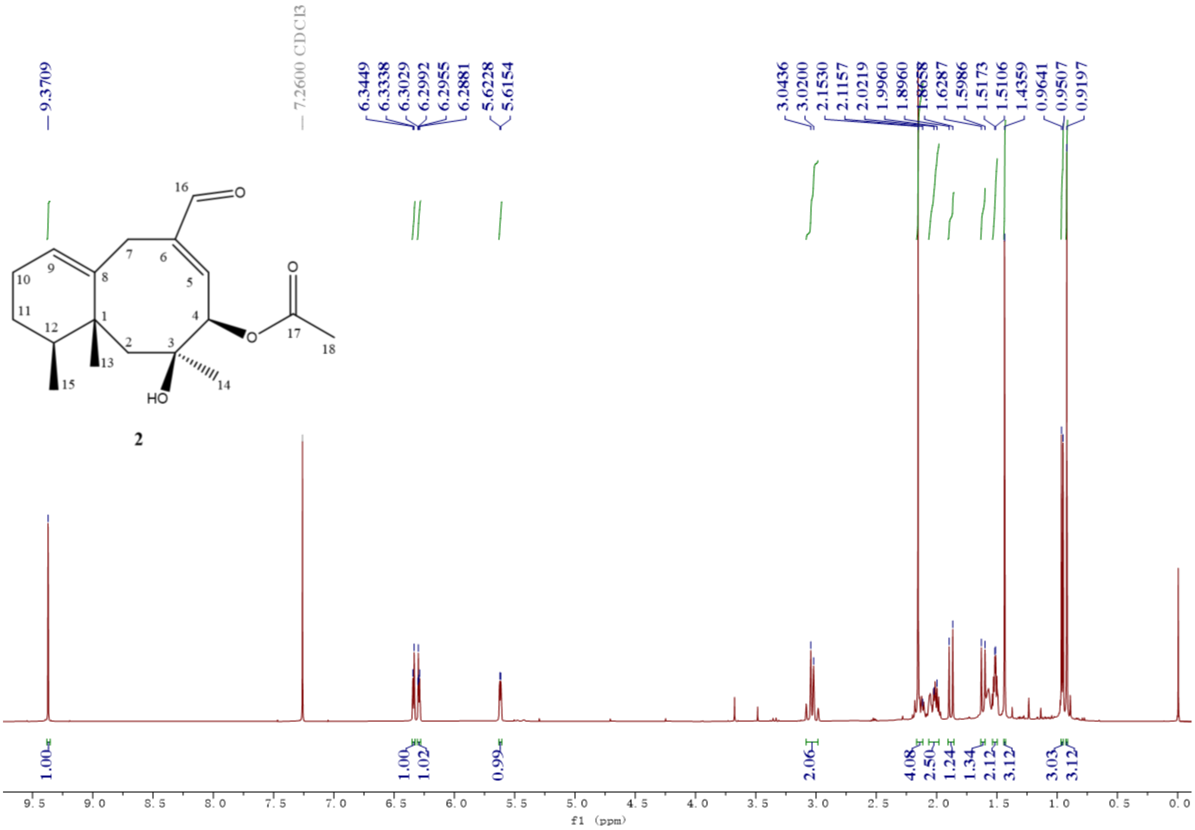

Figure S15. ^{1}H NMR spectrum of compound **2** in $CDCl_3$, 500MHz.

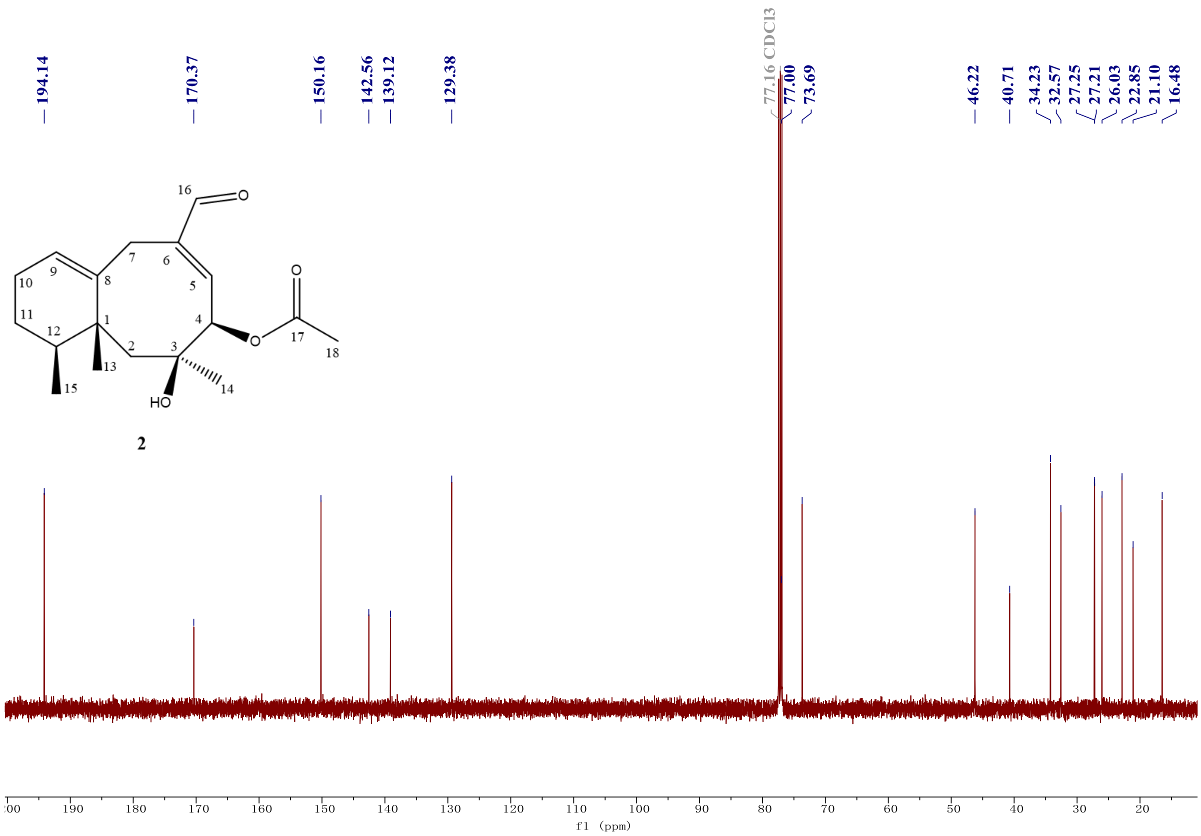

Figure S16. ^{13}C NMR spectrum of compound **2** in $CDCl_3$, 125MHz.

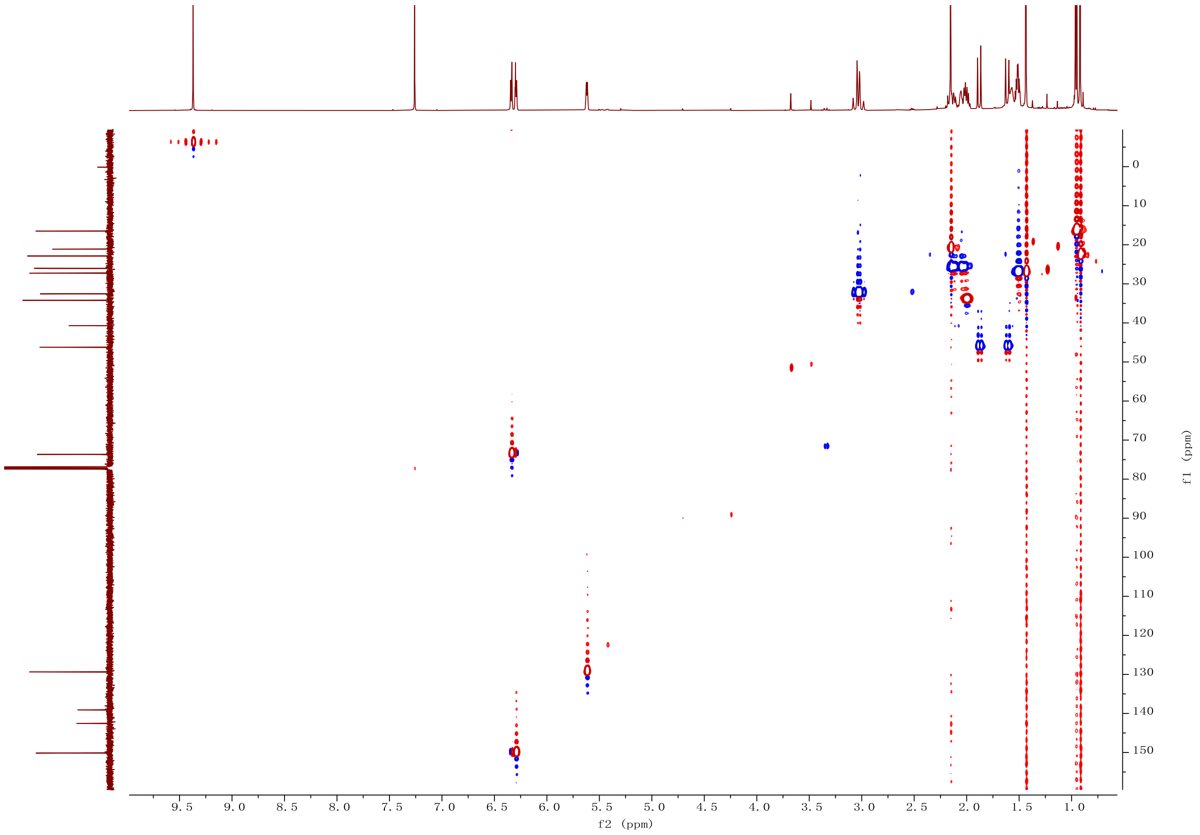

Figure S17. HSQC spectrum of compound **2** in $CDCl_3$, 500MHz.

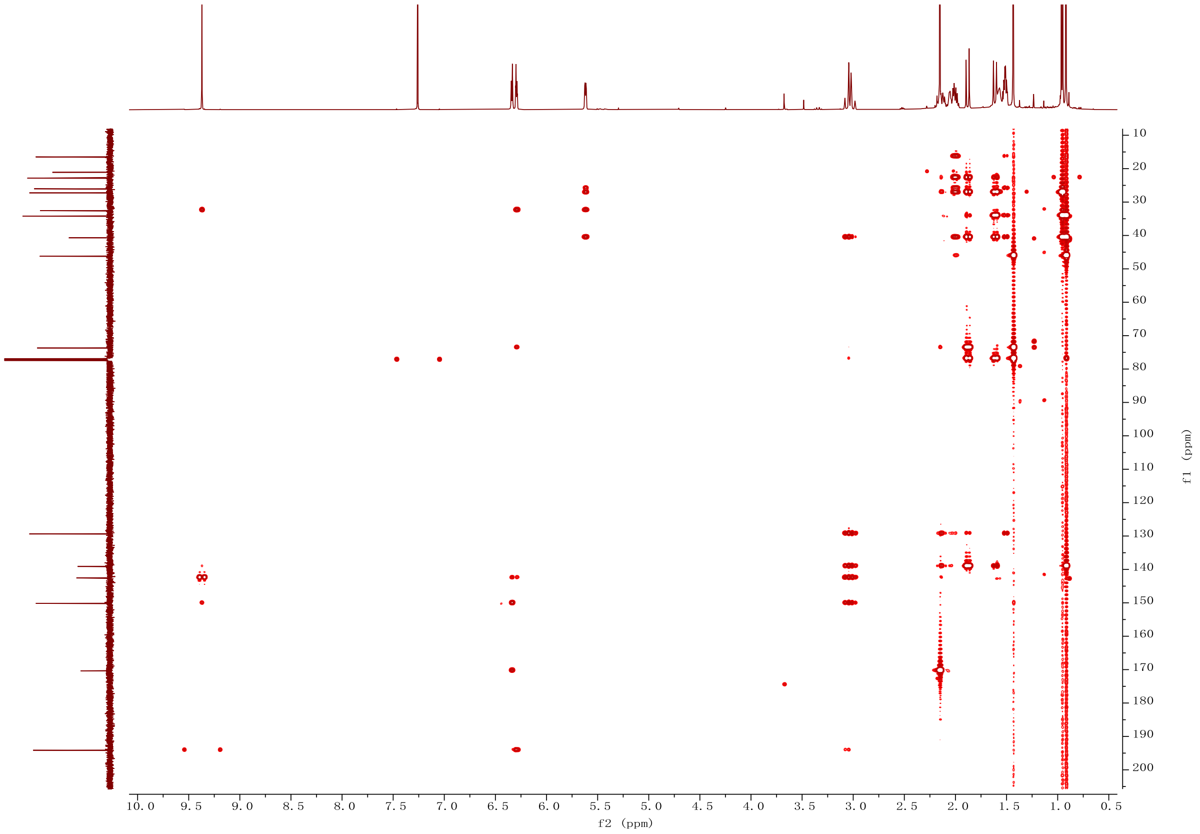

Figure S18. HMBC NMR spectrum of compound **2** in $CDCl_3$, 500MHz.

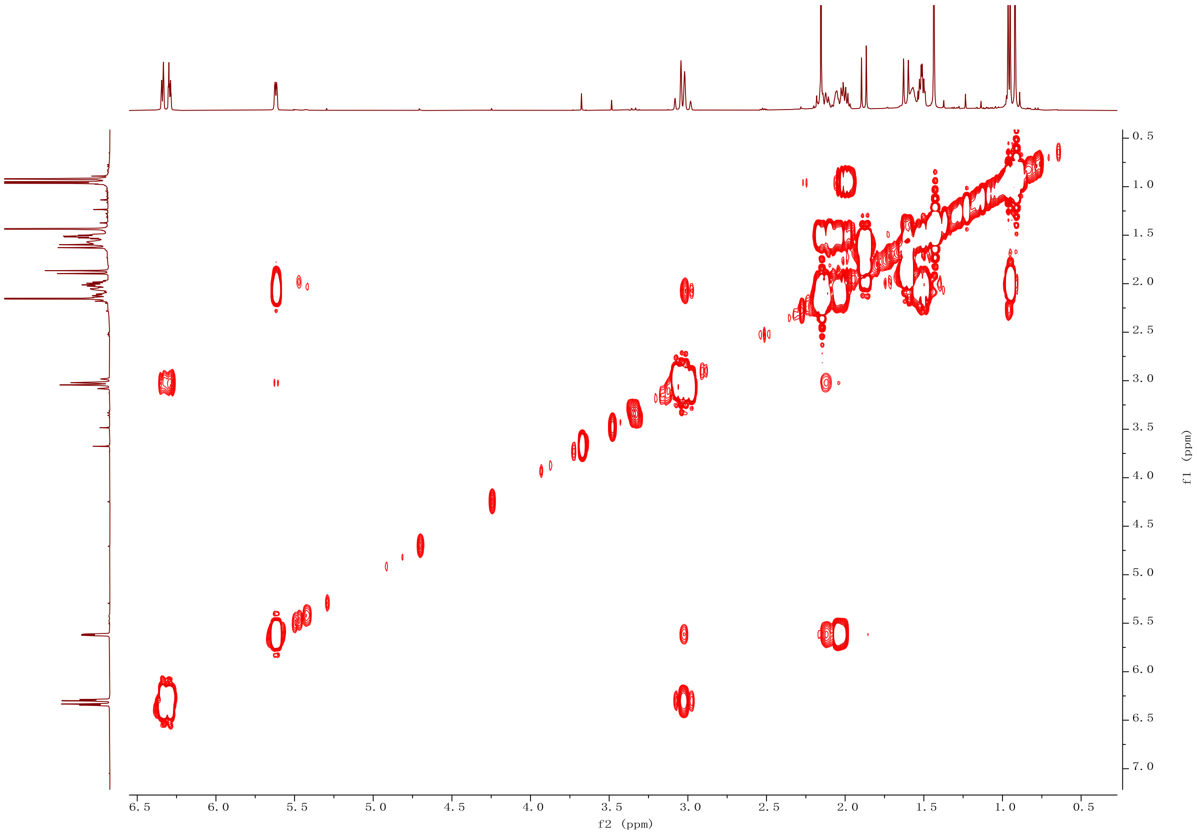

Figure S19. ^{1}H-^{1}H COSY NMR spectrum of compound **2** in $CDCl_3$, 500MHz.

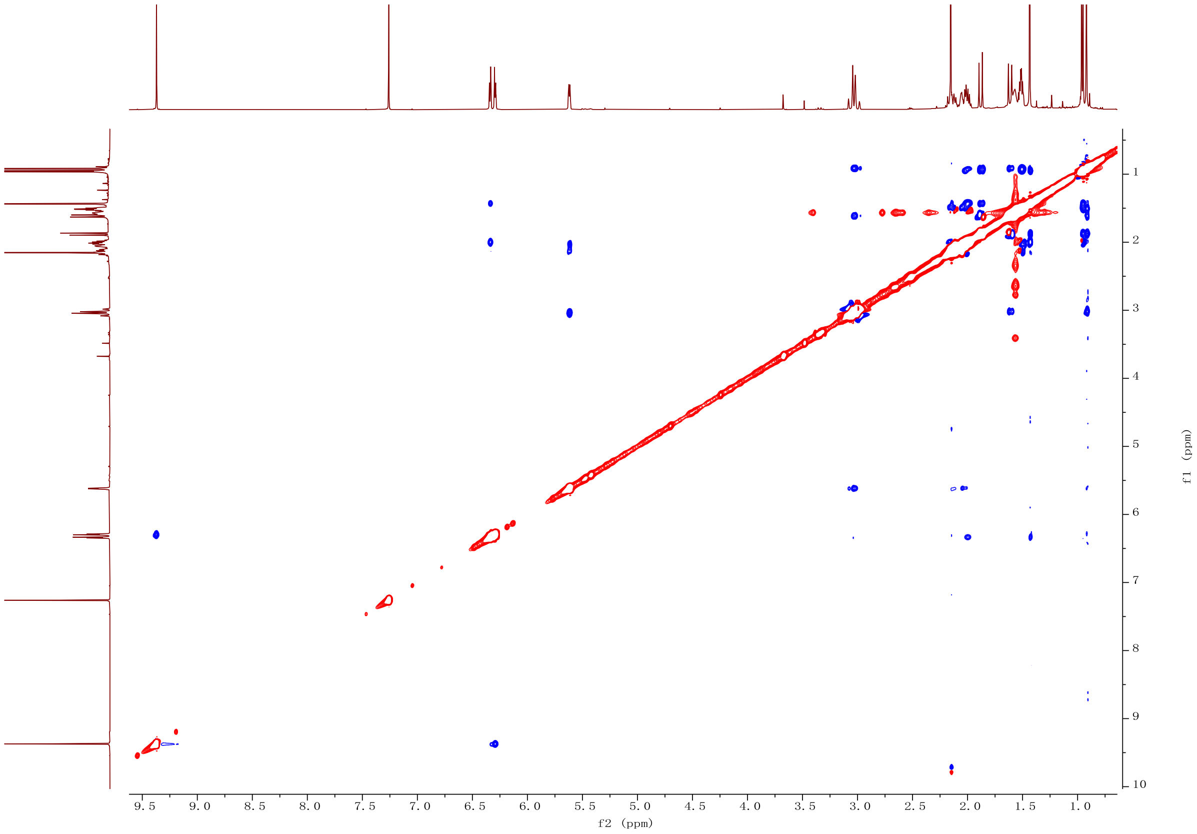

Figure S20. NOESY NMR spectrum of compound **2** in $CDCl_3$, 500MHz.

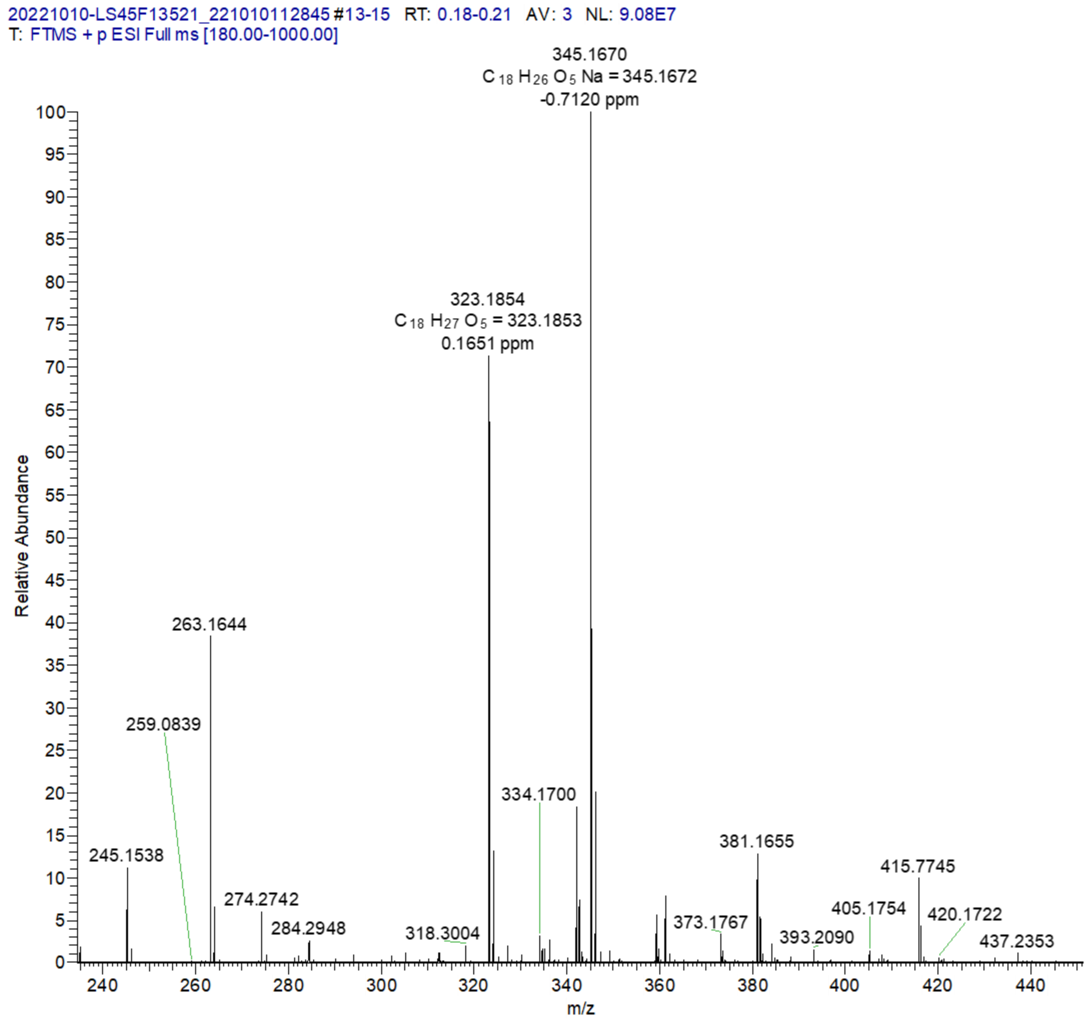

Figure S21. HRESIMS spectrum of compound **3**.

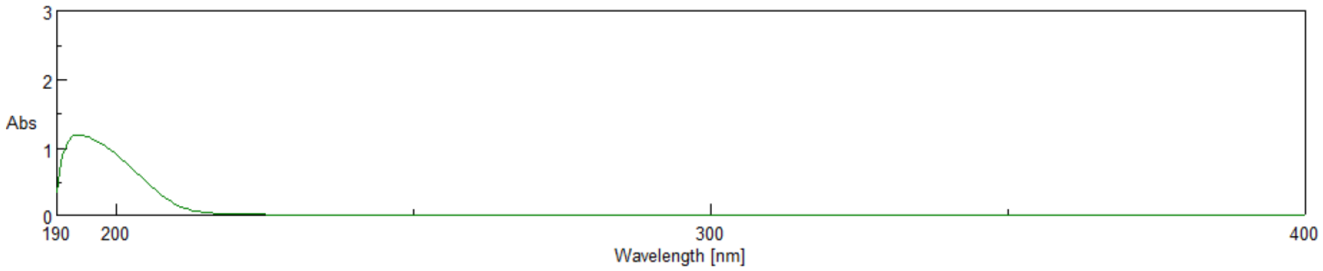

Figure S22. UV spectrum of compound **3**.

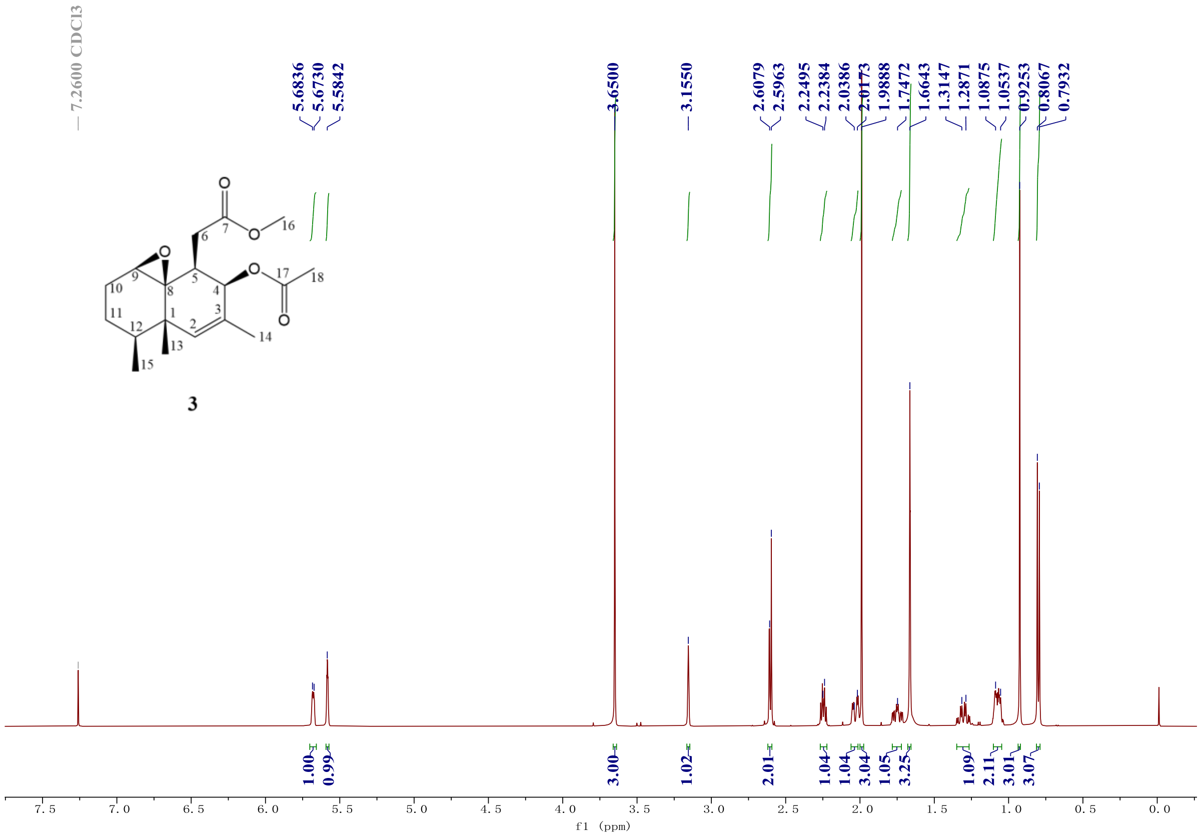

Figure S23. ^{1}H NMR spectrum of compound **3** in $CDCl_3$, 500MHz.

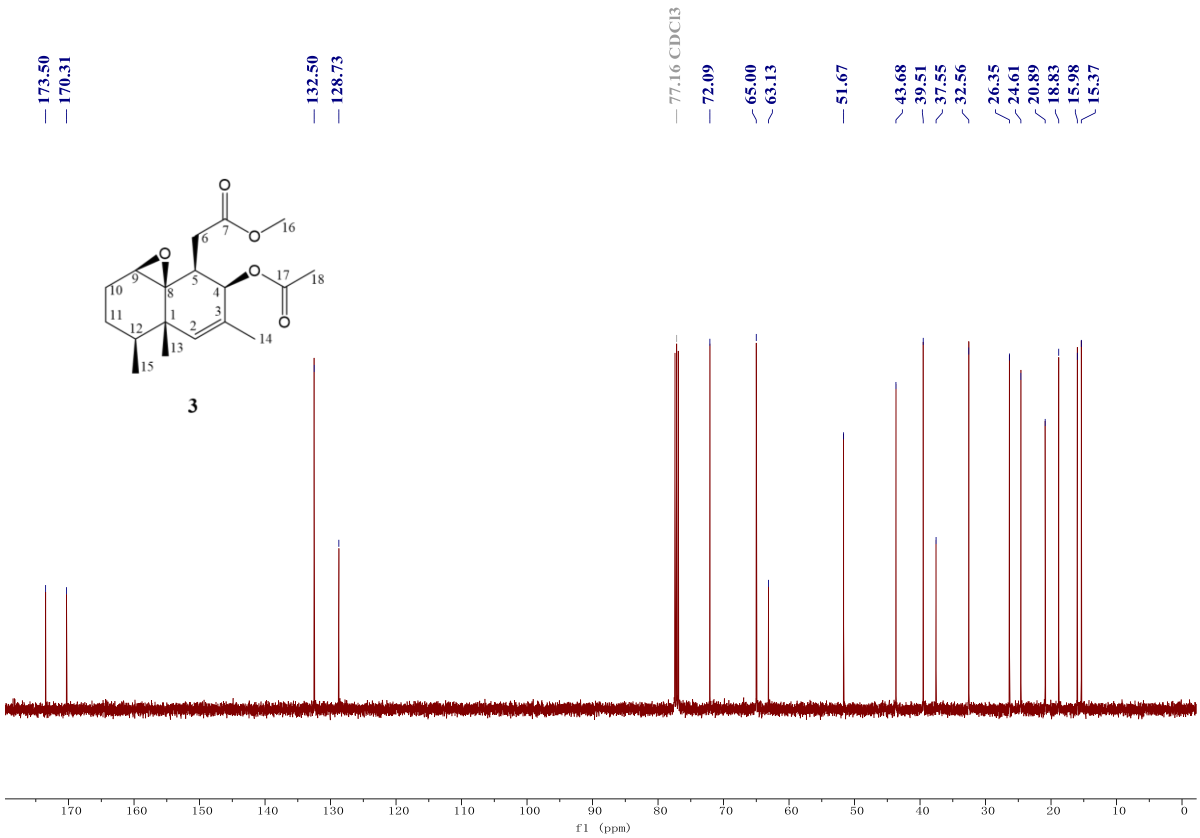

Figure S24. ^{13}C NMR spectrum of compound **3** in $CDCl_3$, 125MHz.

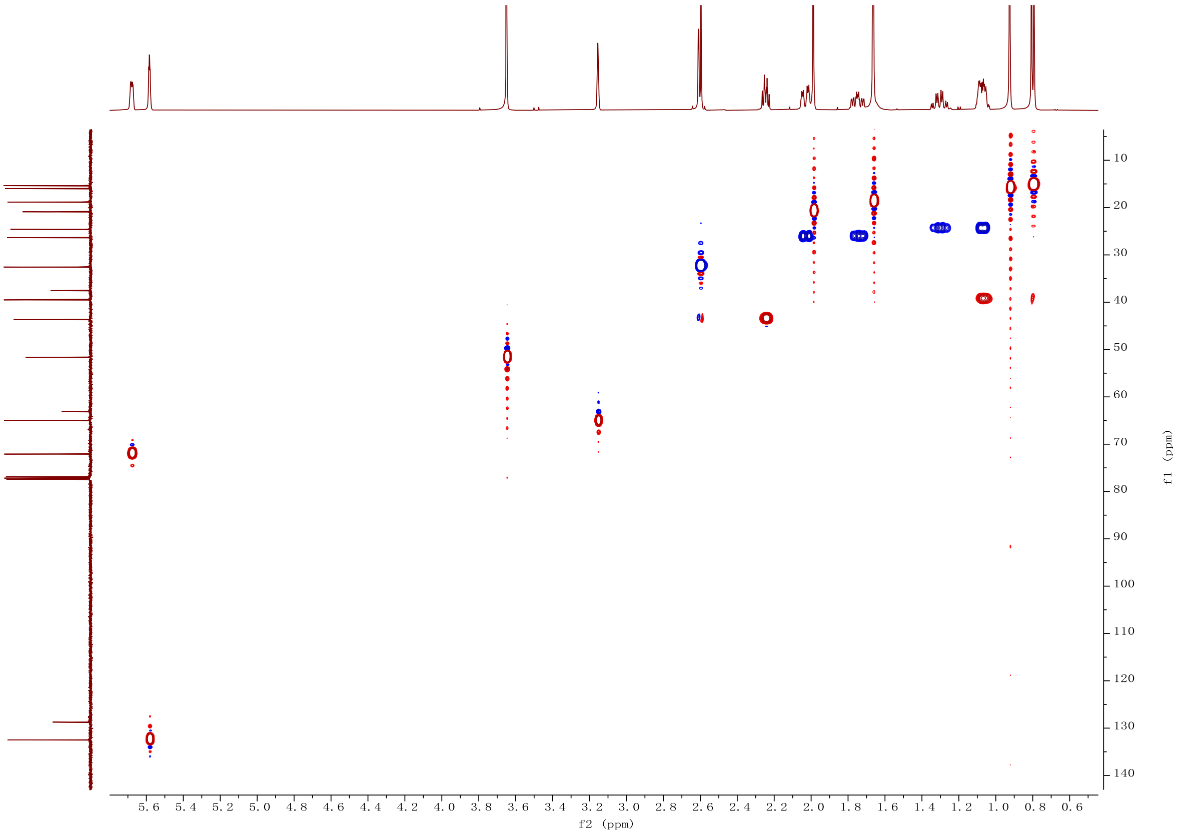

Figure S25. HSQC spectrum of compound **3** in CDCl3, 500MHz.

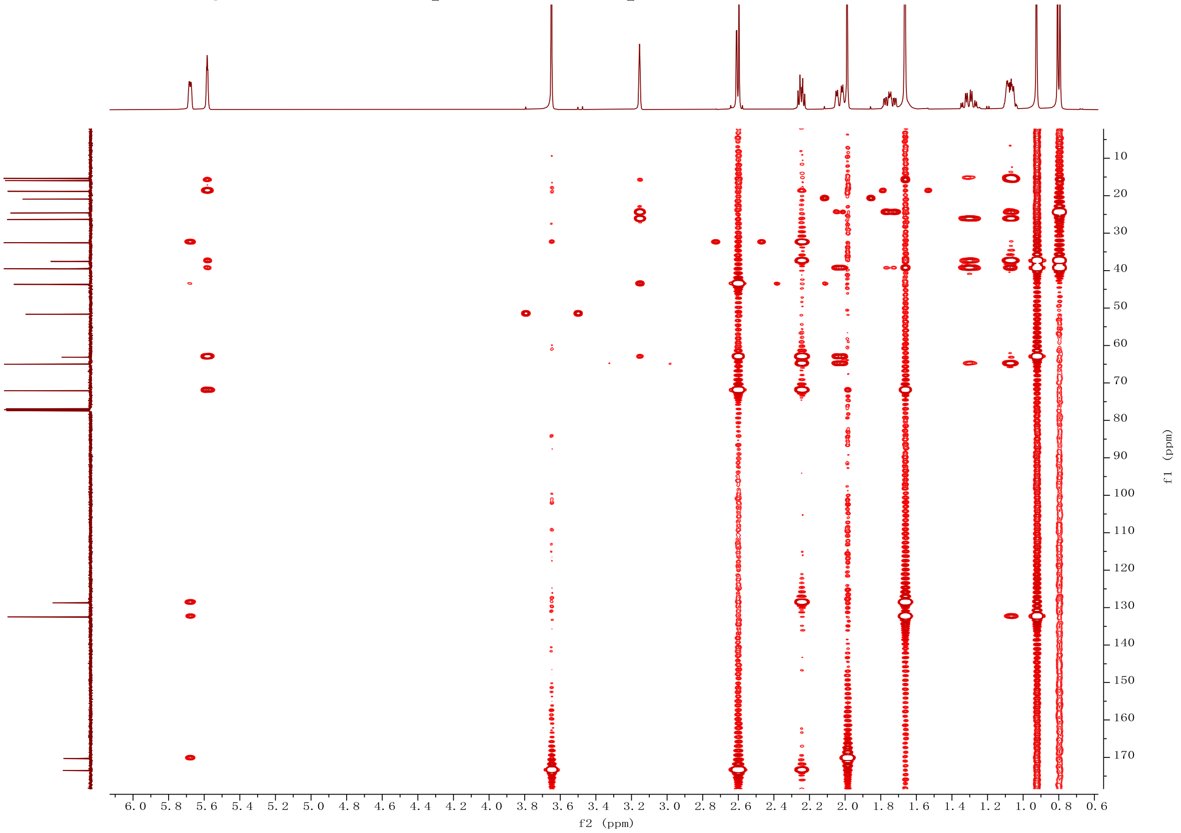

Figure S26. HMBC NMR spectrum of compound **3** in $CDCl_3$, 500MHz.

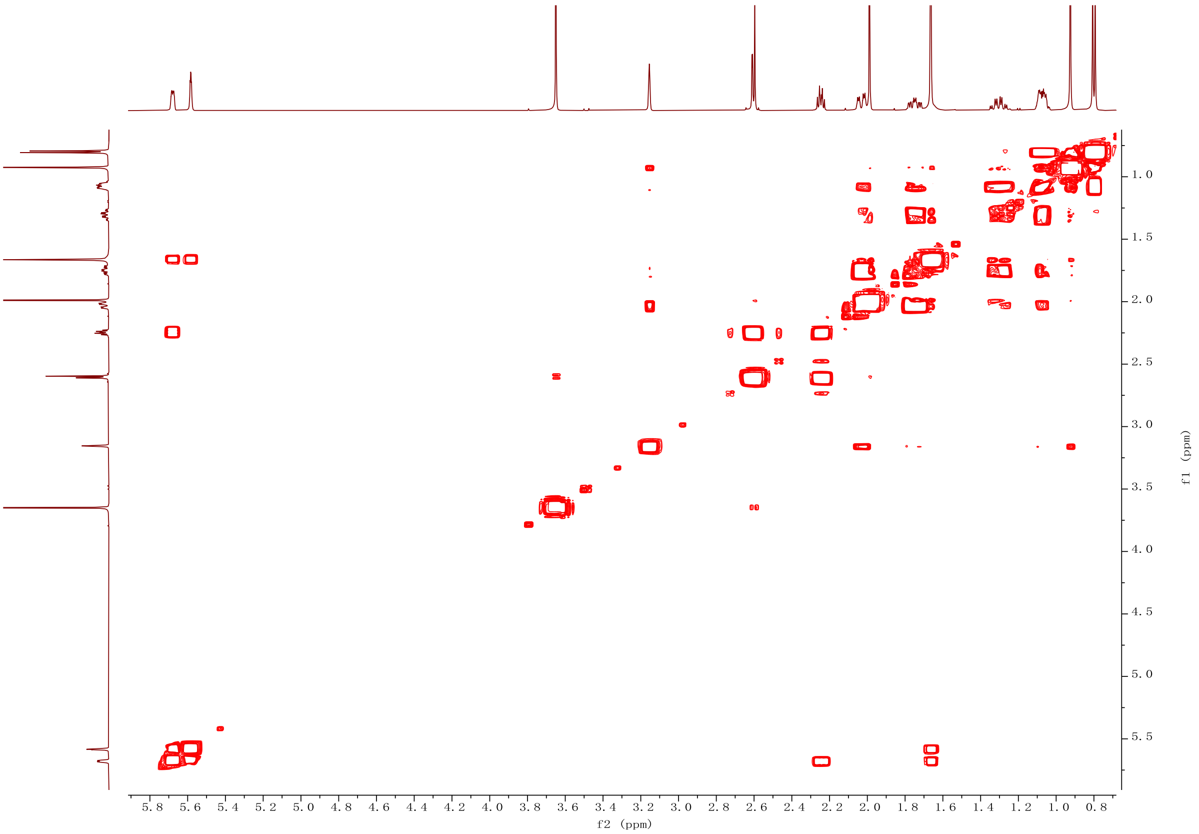

Figure S27. ^1H-^1H COSY NMR spectrum of compound **3** in $CDCl_3$, 500MHz.

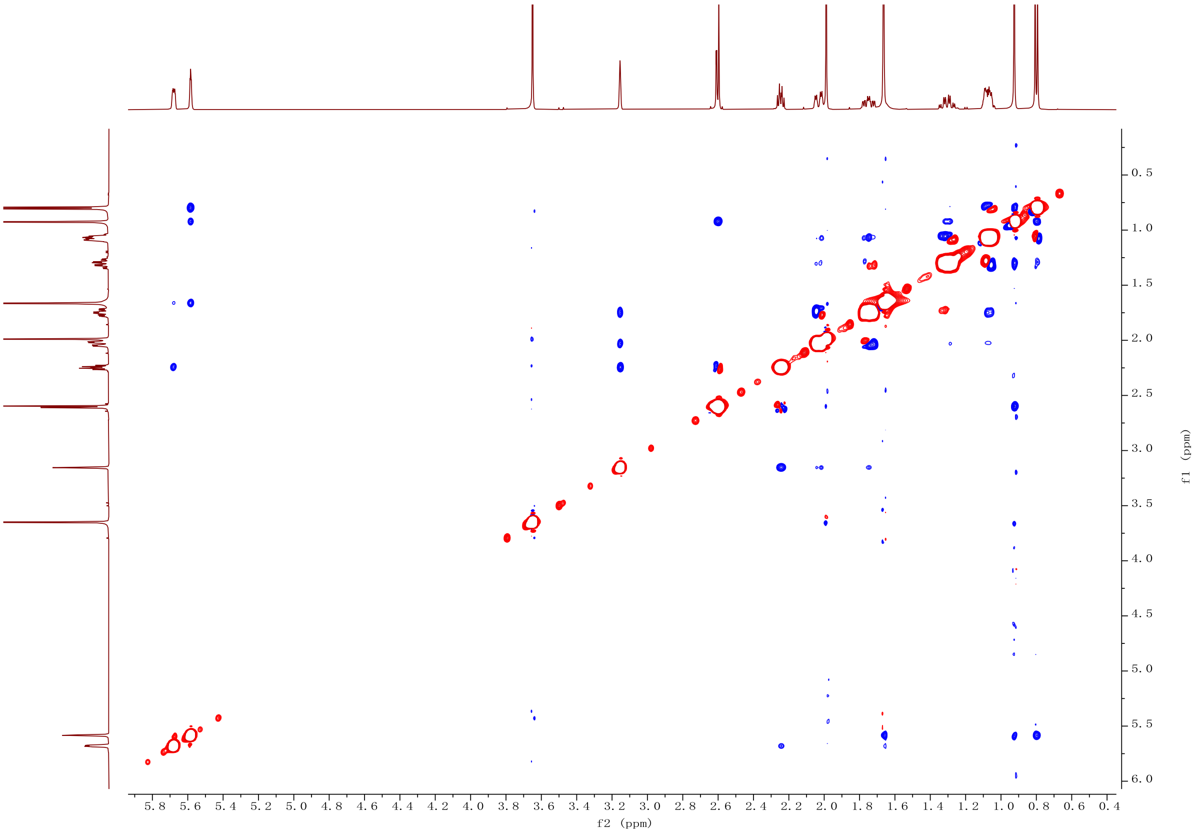

Figure S28. NOESY NMR spectrum of compound **3** in $CDCl_3$, 500MHz.

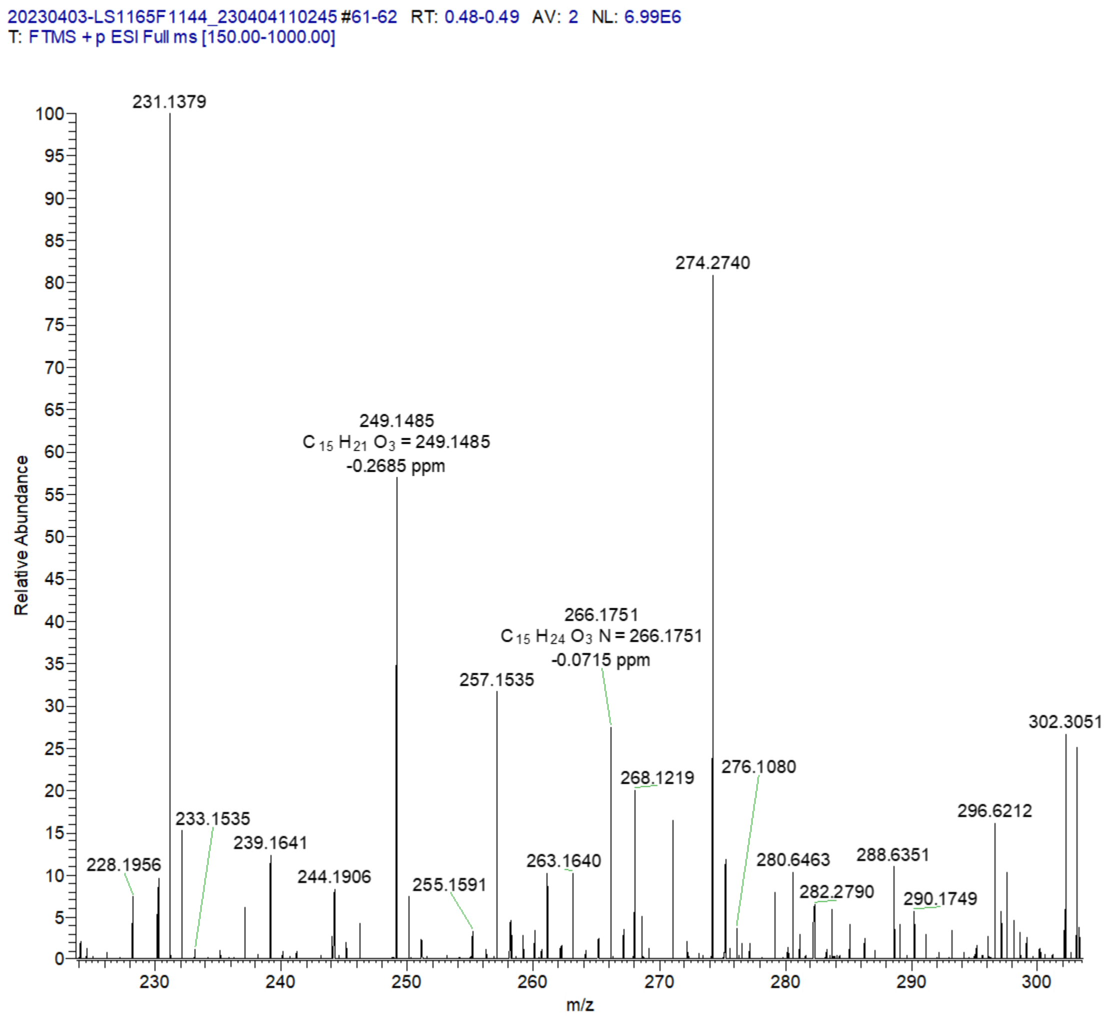

Figure S29. HRESIMS spectrum of compound **4.**

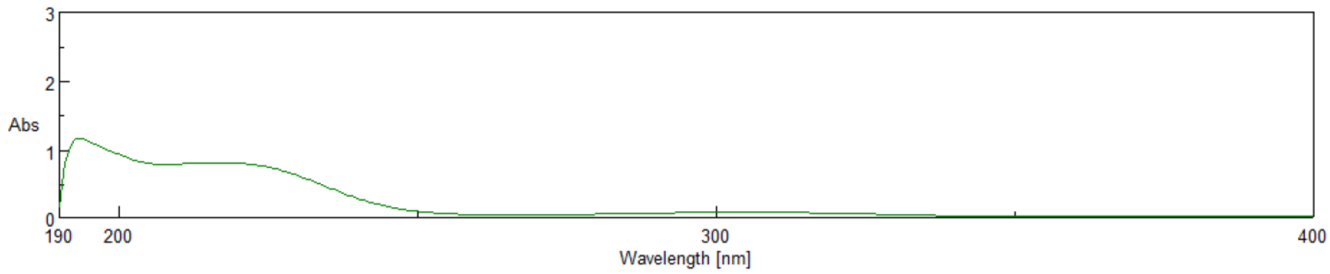

Figure S30. UV spectrum of compound **4**.

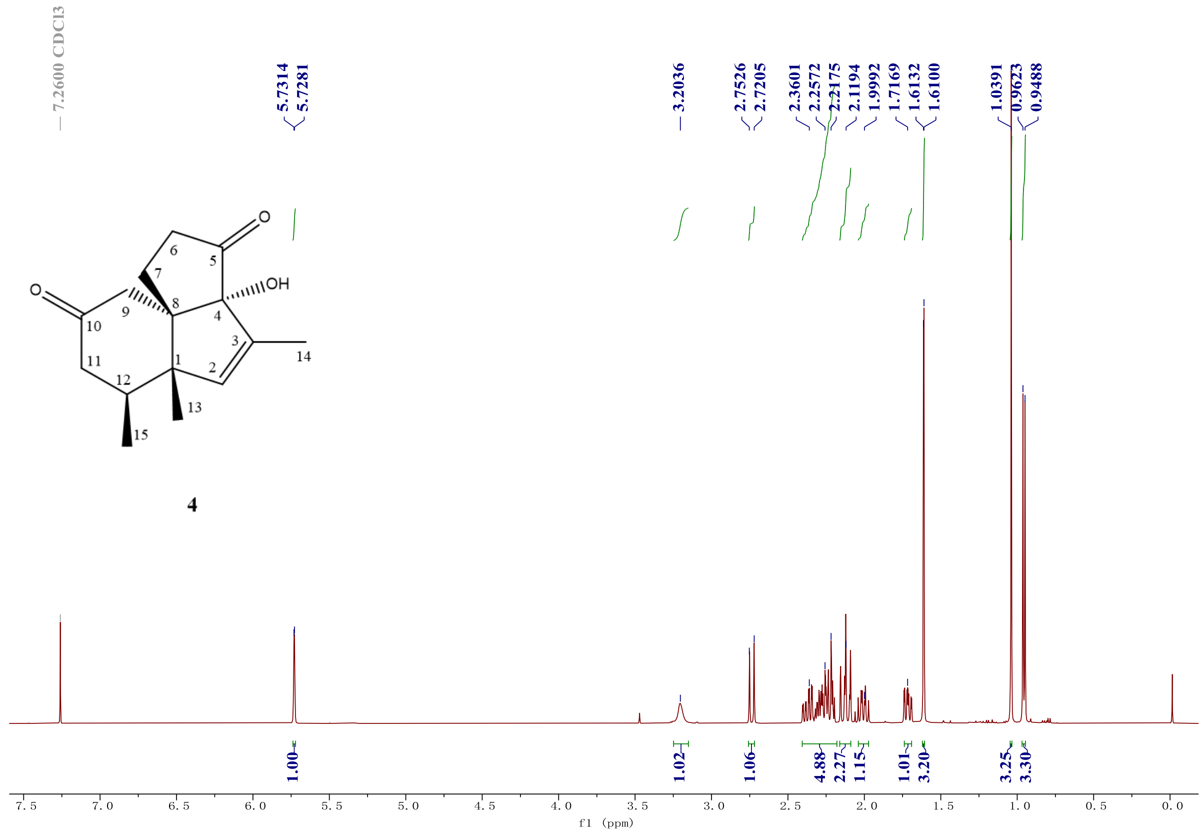

Figure S31. ^{1}H NMR spectrum of compound **4** in $CDCl_3$, 500MHz.

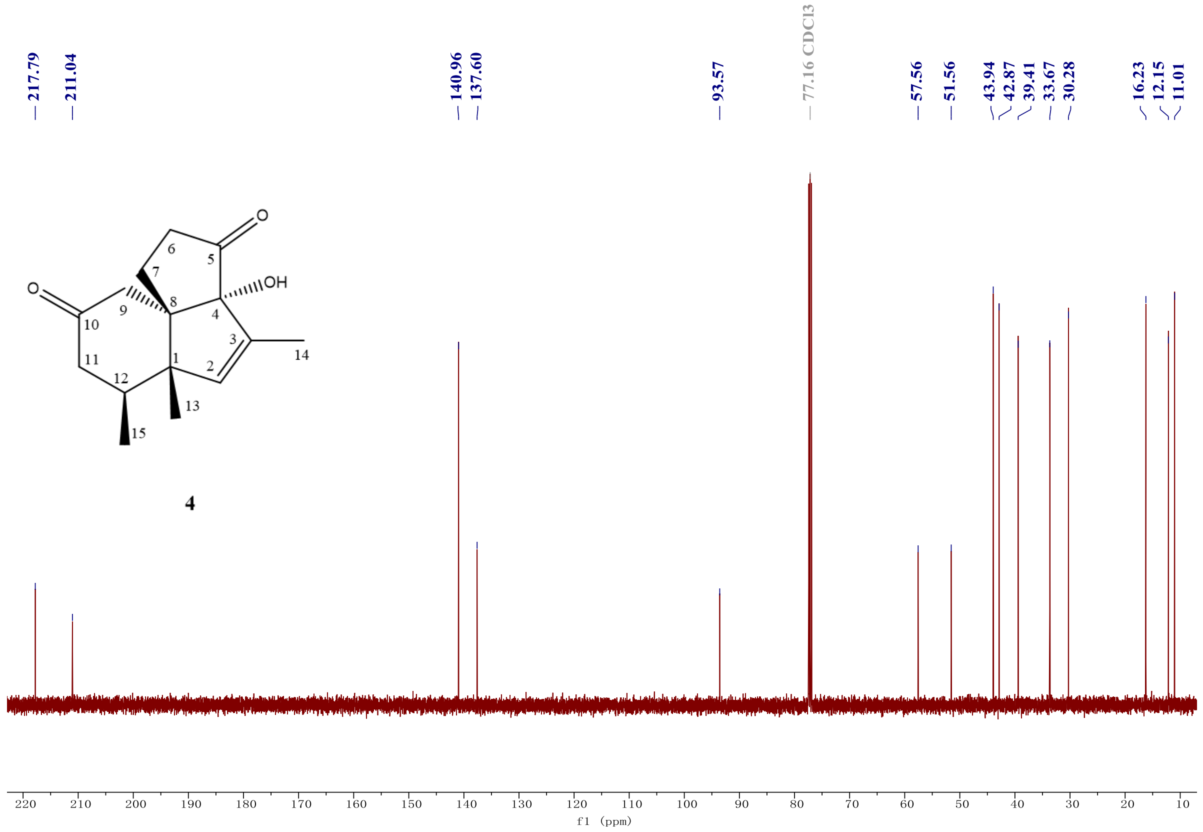

Figure S32. ^{13}C NMR spectrum of compound **4** in $CDCl_3$, 125MHz.

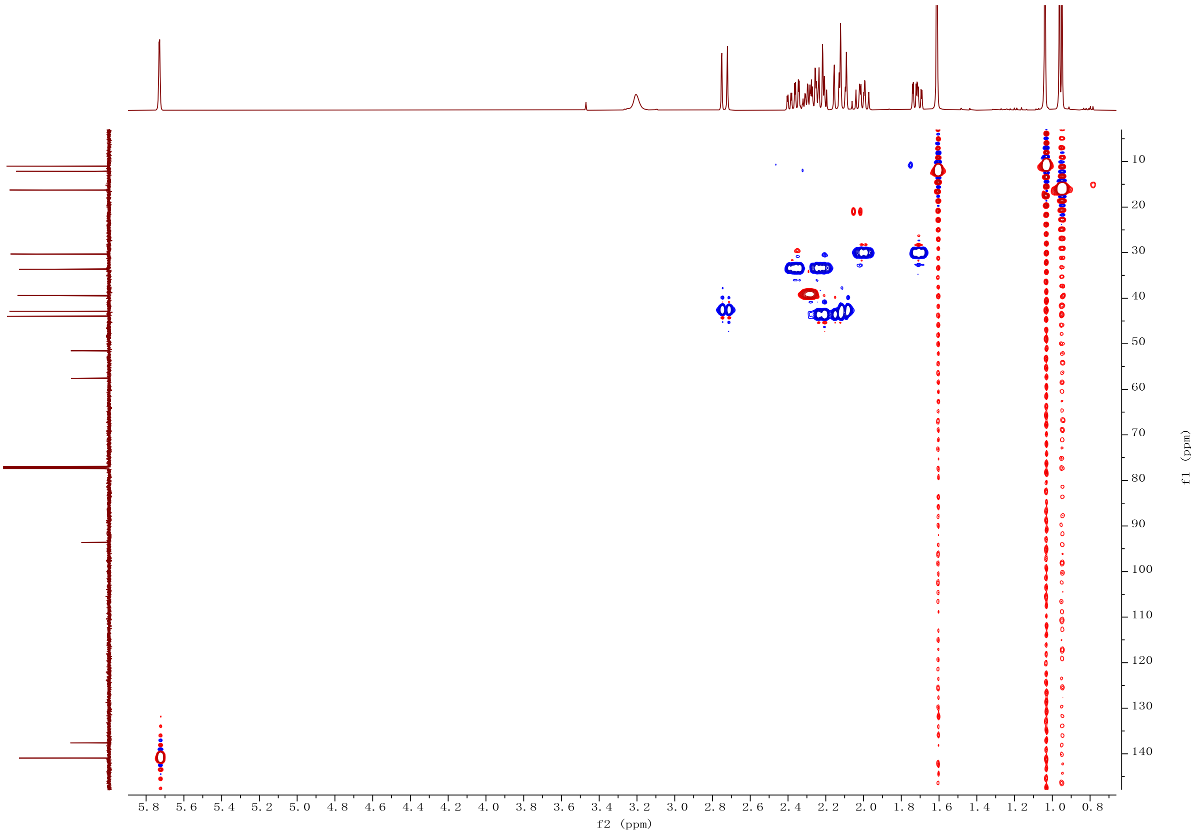

Figure S33. HSQC spectrum of compound **4** in $CDCl_3$, 500MHz.

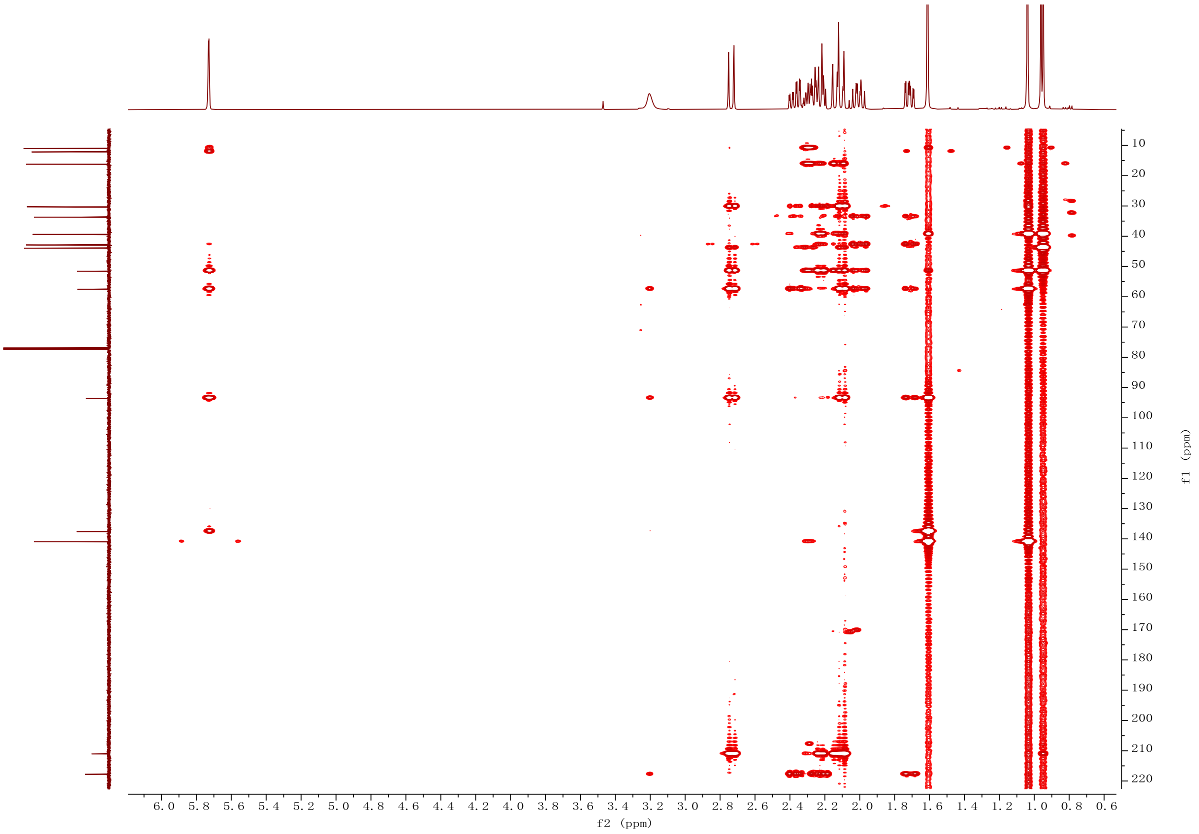

Figure S34. HMBC NMR spectrum of compound **4** in $CDCl_3$, 500MHz.

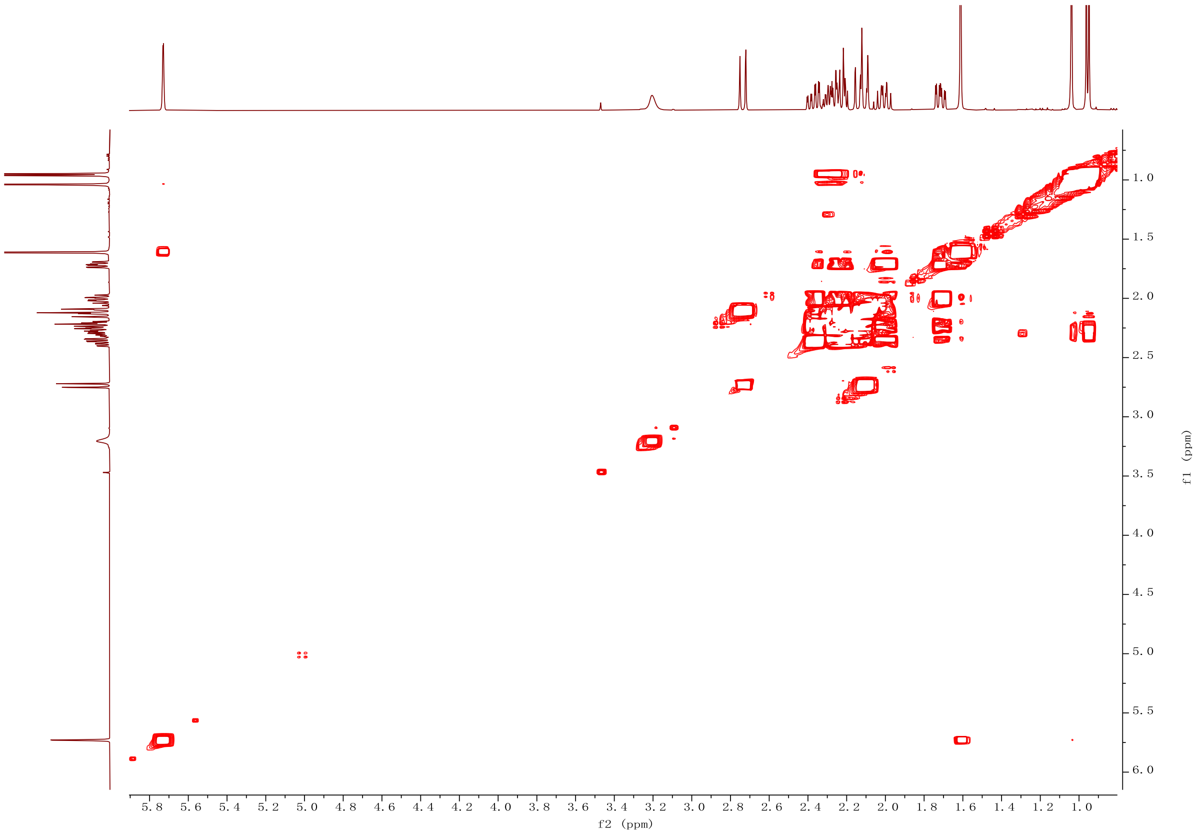

Figure S35. ^1H-^1H COSY NMR spectrum of compound **4** in $CDCl_3$, 500MHz.

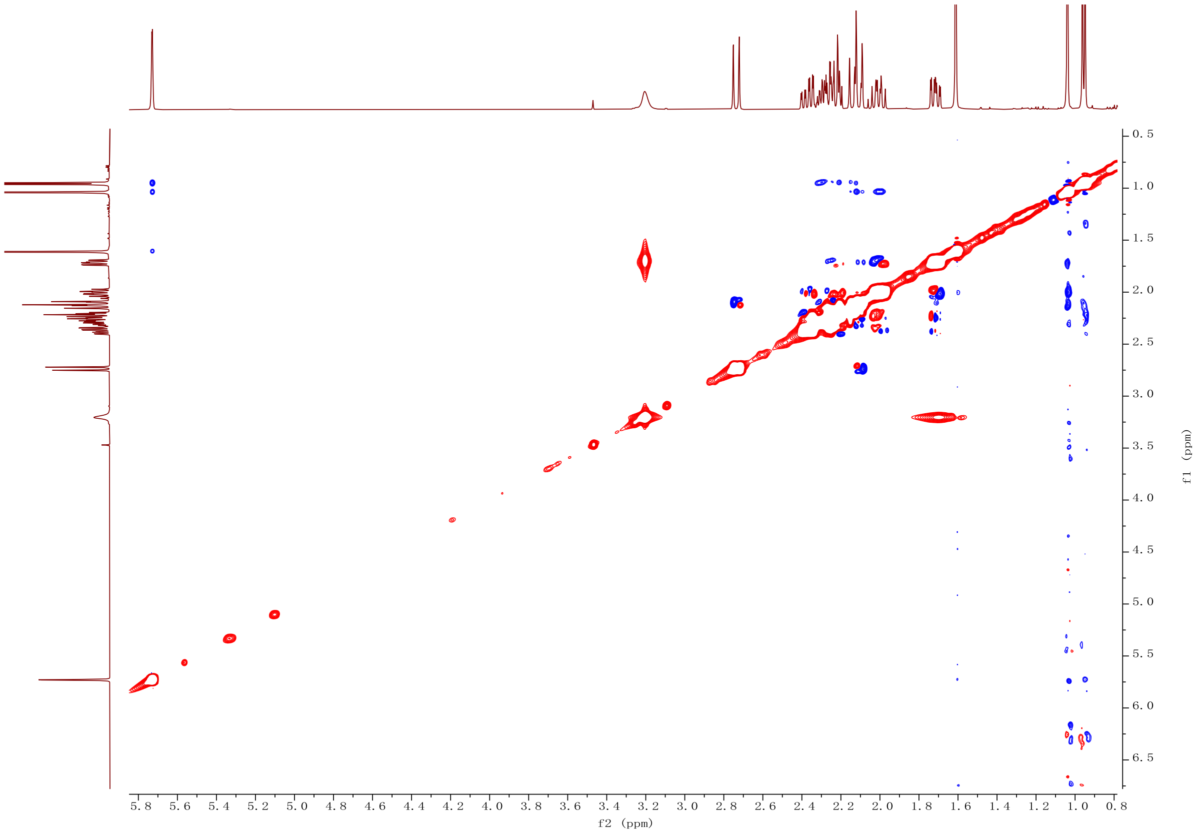

Figure S36. NOESY NMR spectrum of compound **4** in $CDCl_3$, 500MHz.

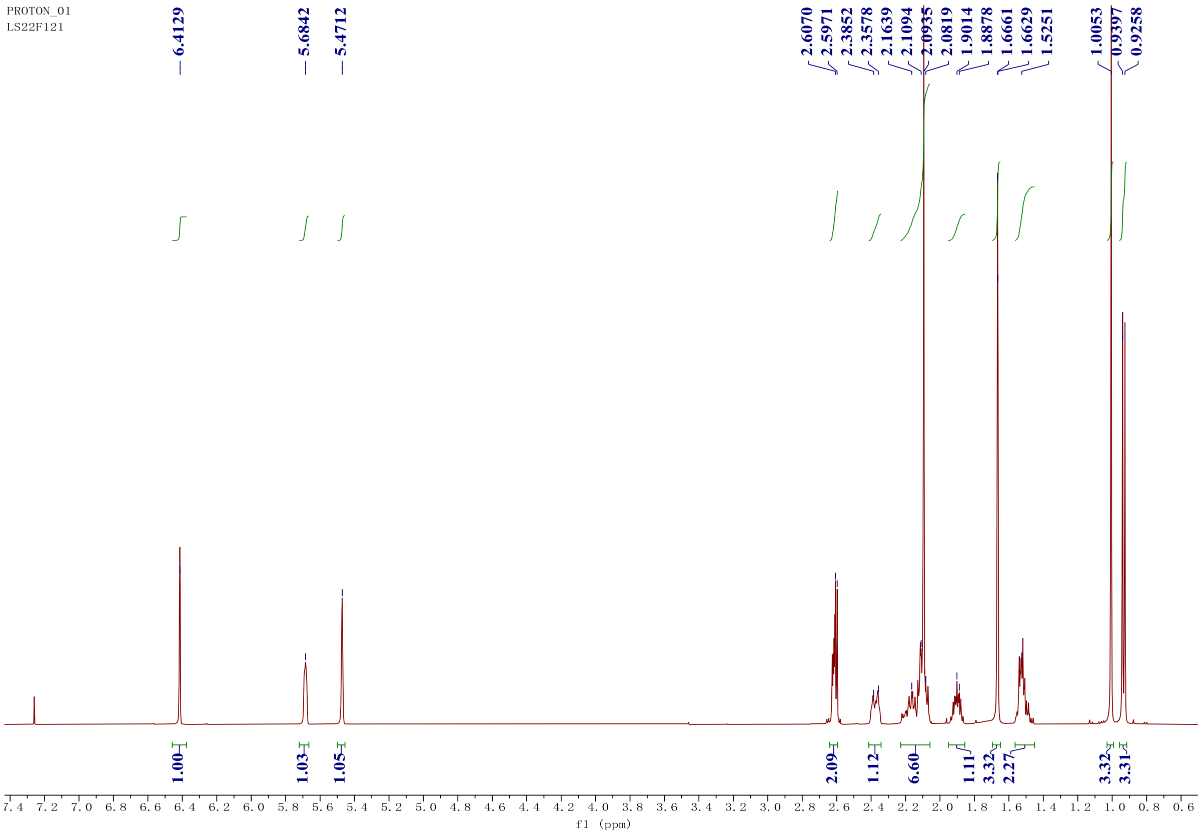

Figure S37. ^{1}H NMR spectrum of compound **5** in $CDCl_3$, 500MHz.

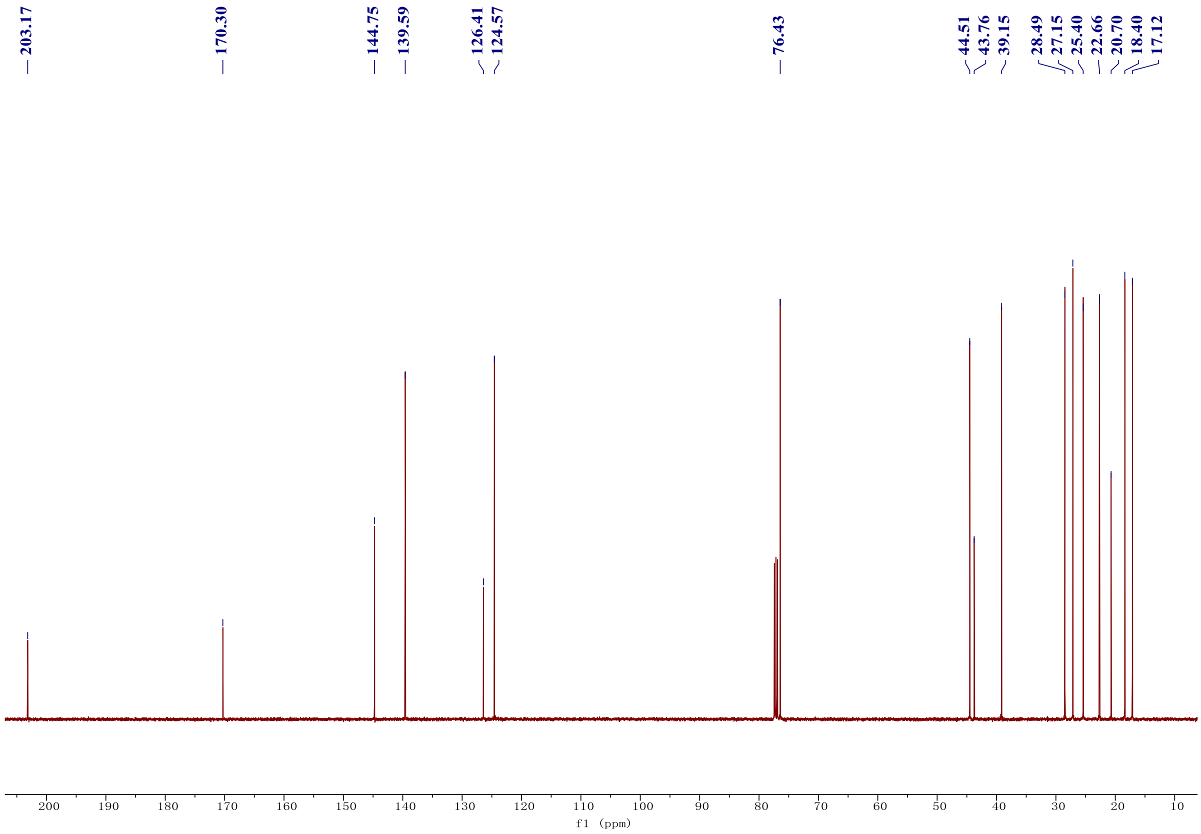

Figure S38. ^{13}C NMR spectrum of compound **5** in $CDCl_3$, 125MHz.

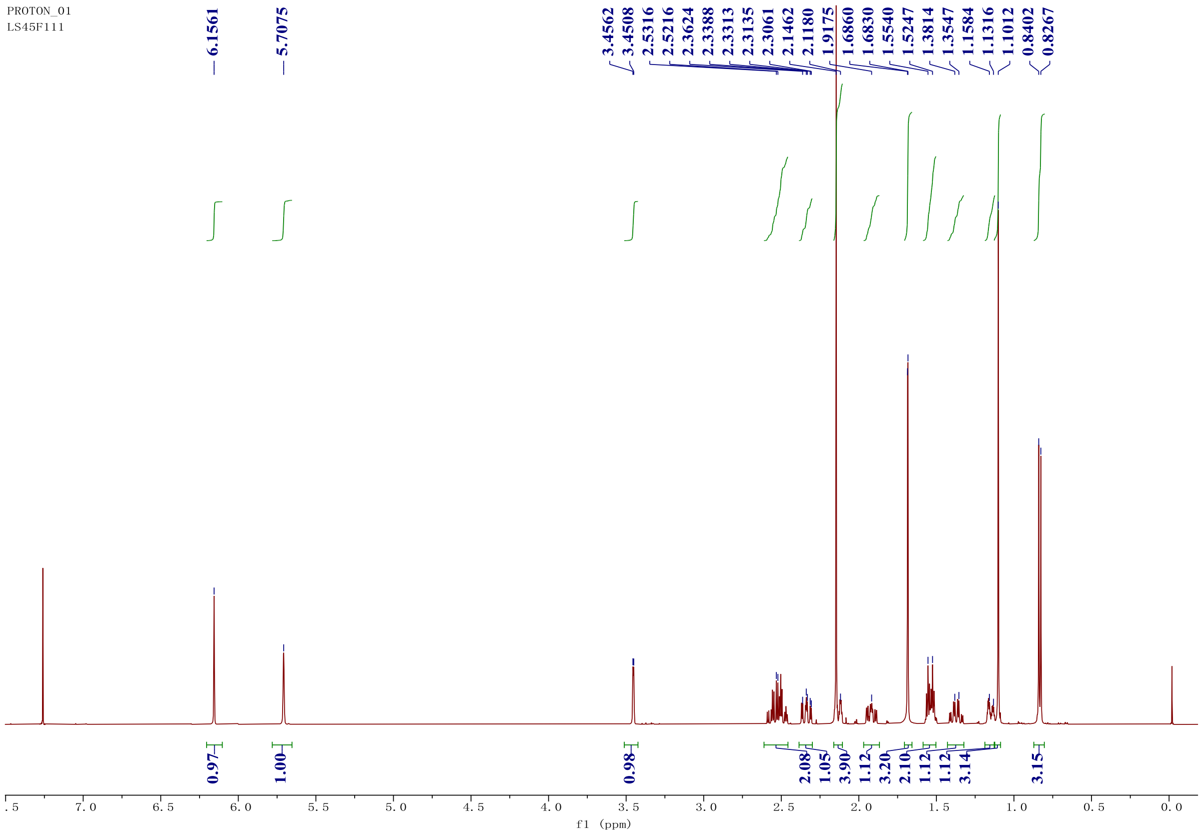

Figure S39. ^{1}H NMR spectrum of compound **6** in $CDCl_3$, 500MHz.

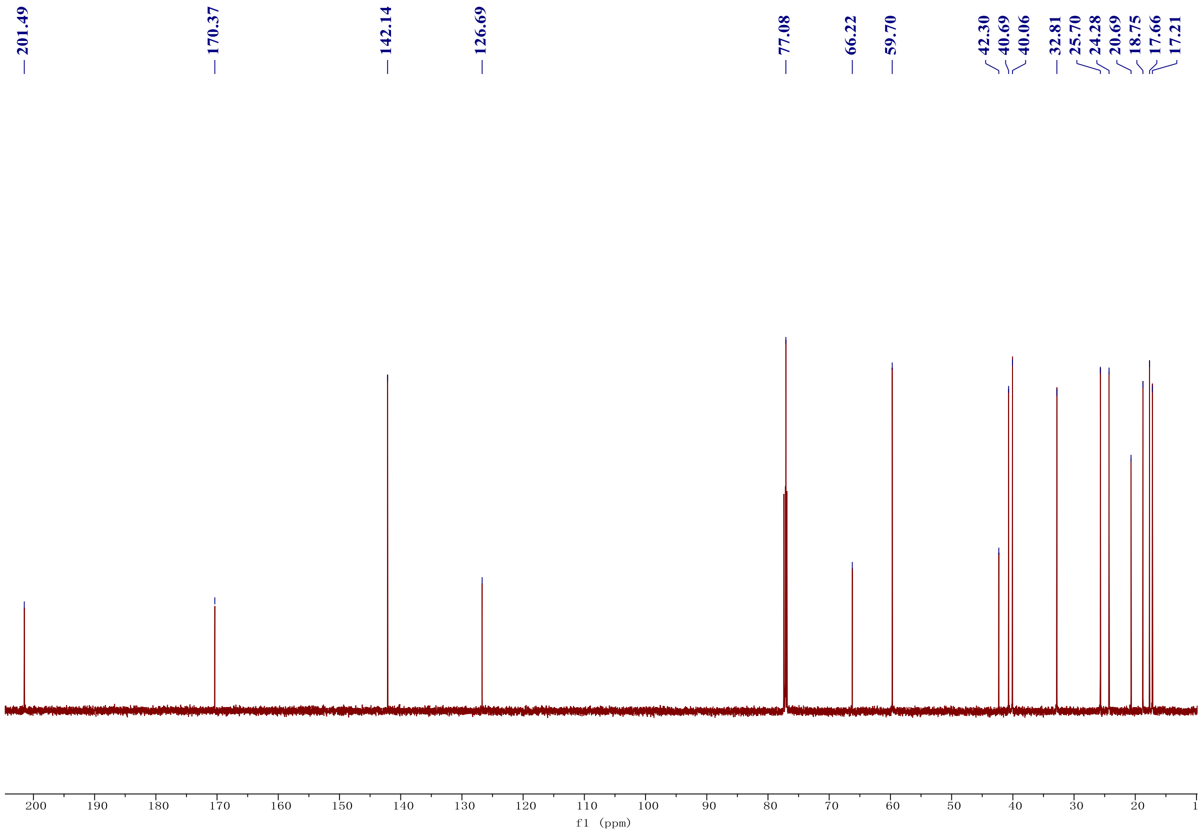

Figure S40. ^{13}C NMR spectrum of compound **6** in $CDCl_3$, 125MHz.

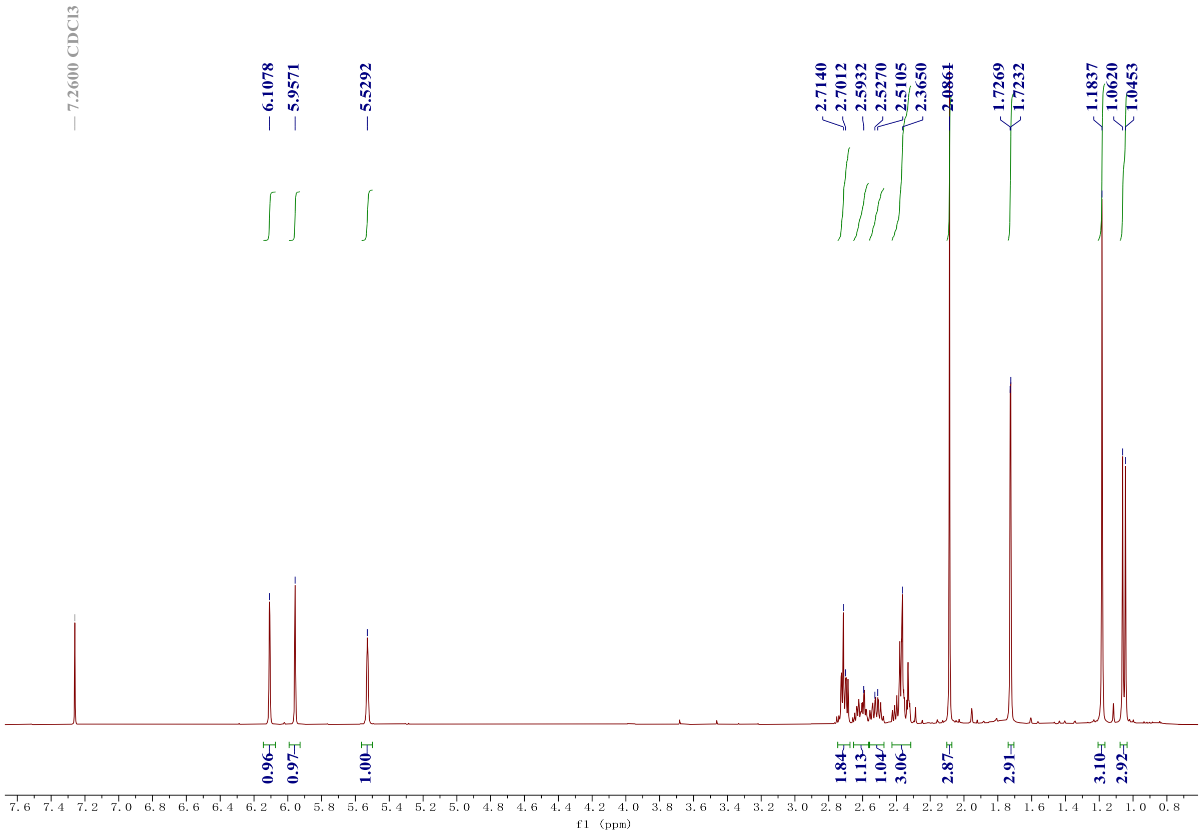

Figure S41. ^{1}H NMR spectrum of compound **7** in $CDCl_3$, 400MHz.

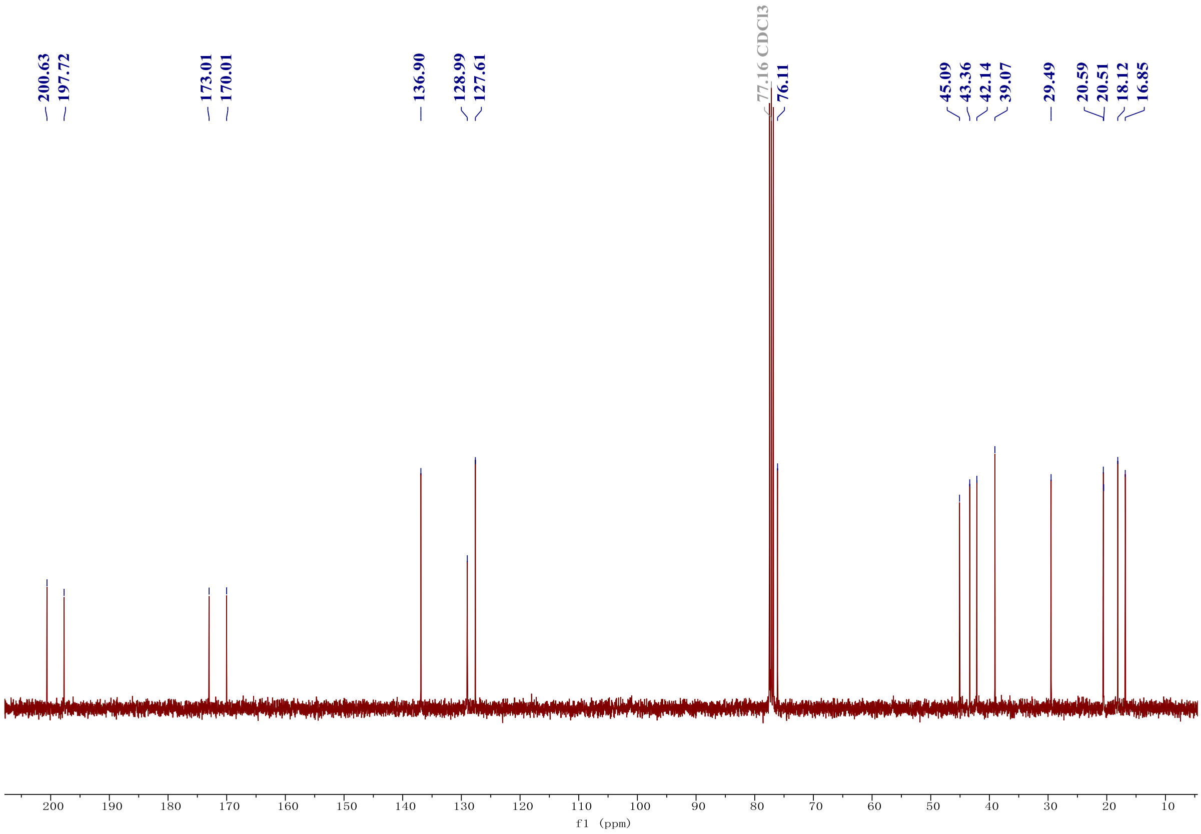

Figure S42. ^{13}C NMR spectrum of compound **7** in $CDCl_3$, 100MHz.
